# DNA methylation clocks for estimating biological age in Chinese cohorts

**DOI:** 10.1093/procel/pwae011

**Published:** 2024-03-14

**Authors:** Zikai Zheng, Jiaming Li, Tianzi Liu, Yanling Fan, Qiao-Cheng Zhai, Muzhao Xiong, Qiao-Ran Wang, Xiaoyan Sun, Qi-Wen Zheng, Shanshan Che, Beier Jiang, Quan Zheng, Cui Wang, Lixiao Liu, Jiale Ping, Si Wang, Dan-Dan Gao, Jinlin Ye, Kuan Yang, Yuesheng Zuo, Shuai Ma, Yun-Gui Yang, Jing Qu, Feng Zhang, Peilin Jia, Guang-Hui Liu, Weiqi Zhang

**Affiliations:** CAS Key Laboratory of Genomic and Precision Medicine, Beijing Institute of Genomics, Chinese Academy of Sciences and China National Center for Bioinformation, Beijing 100101, China; University of Chinese Academy of Sciences, Beijing 100049, China; CAS Key Laboratory of Genomic and Precision Medicine, Beijing Institute of Genomics, Chinese Academy of Sciences and China National Center for Bioinformation, Beijing 100101, China; University of Chinese Academy of Sciences, Beijing 100049, China; CAS Key Laboratory of Genomic and Precision Medicine, Beijing Institute of Genomics, Chinese Academy of Sciences and China National Center for Bioinformation, Beijing 100101, China; CAS Key Laboratory of Computational Biology, Shanghai Institute of Nutrition and Health, Chinese Academy of Sciences, Shanghai 200031, China; CAS Key Laboratory of Genomic and Precision Medicine, Beijing Institute of Genomics, Chinese Academy of Sciences and China National Center for Bioinformation, Beijing 100101, China; Division of Orthopaedics, Quzhou Affiliated Hospital of Wenzhou Medical University, Quzhou 324000, China; The Joint Innovation Center for Engineering in Medicine, Quzhou Affiliated Hospital of Wenzhou Medical University, Quzhou 324000, China; CAS Key Laboratory of Genomic and Precision Medicine, Beijing Institute of Genomics, Chinese Academy of Sciences and China National Center for Bioinformation, Beijing 100101, China; University of Chinese Academy of Sciences, Beijing 100049, China; CAS Key Laboratory of Genomic and Precision Medicine, Beijing Institute of Genomics, Chinese Academy of Sciences and China National Center for Bioinformation, Beijing 100101, China; University of Chinese Academy of Sciences, Beijing 100049, China; CAS Key Laboratory of Genomic and Precision Medicine, Beijing Institute of Genomics, Chinese Academy of Sciences and China National Center for Bioinformation, Beijing 100101, China; University of Chinese Academy of Sciences, Beijing 100049, China; CAS Key Laboratory of Genomic and Precision Medicine, Beijing Institute of Genomics, Chinese Academy of Sciences and China National Center for Bioinformation, Beijing 100101, China; CAS Key Laboratory of Genomic and Precision Medicine, Beijing Institute of Genomics, Chinese Academy of Sciences and China National Center for Bioinformation, Beijing 100101, China; University of Chinese Academy of Sciences, Beijing 100049, China; The Joint Innovation Center for Engineering in Medicine, Quzhou Affiliated Hospital of Wenzhou Medical University, Quzhou 324000, China; The Joint Innovation Center for Engineering in Medicine, Quzhou Affiliated Hospital of Wenzhou Medical University, Quzhou 324000, China; CAS Key Laboratory of Genomic and Precision Medicine, Beijing Institute of Genomics, Chinese Academy of Sciences and China National Center for Bioinformation, Beijing 100101, China; University of Chinese Academy of Sciences, Beijing 100049, China; CAS Key Laboratory of Genomic and Precision Medicine, Beijing Institute of Genomics, Chinese Academy of Sciences and China National Center for Bioinformation, Beijing 100101, China; University of Chinese Academy of Sciences, Beijing 100049, China; CAS Key Laboratory of Genomic and Precision Medicine, Beijing Institute of Genomics, Chinese Academy of Sciences and China National Center for Bioinformation, Beijing 100101, China; University of Chinese Academy of Sciences, Beijing 100049, China; Advanced Innovation Center for Human Brain Protection, and National Clinical Research Center for Geriatric Disorders, Xuanwu Hospital Capital Medical University, Beijing 100053, China; Aging Translational Medicine Center, International Center for Aging and Cancer, Xuanwu Hospital, Capital Medical University, Beijing 100053, China; Aging Biomarker Consortium, Beijing 100101, China; The Joint Innovation Center for Engineering in Medicine, Quzhou Affiliated Hospital of Wenzhou Medical University, Quzhou 324000, China; The Joint Innovation Center for Engineering in Medicine, Quzhou Affiliated Hospital of Wenzhou Medical University, Quzhou 324000, China; CAS Key Laboratory of Genomic and Precision Medicine, Beijing Institute of Genomics, Chinese Academy of Sciences and China National Center for Bioinformation, Beijing 100101, China; University of Chinese Academy of Sciences, Beijing 100049, China; CAS Key Laboratory of Genomic and Precision Medicine, Beijing Institute of Genomics, Chinese Academy of Sciences and China National Center for Bioinformation, Beijing 100101, China; University of Chinese Academy of Sciences, Beijing 100049, China; Aging Biomarker Consortium, Beijing 100101, China; State Key Laboratory of Membrane Biology, Institute of Zoology, Chinese Academy of Sciences, Beijing 100101, China; Key Laboratory of Organ Regeneration and Reconstruction, Institute of Zoology, Chinese Academy of Sciences, Beijing 100101, China; Institute for Stem Cell and Regeneration, Chinese Academy of Sciences, Beijing 100101, China; Beijing Institute for Stem Cell and Regenerative Medicine, Beijing 100101, China; CAS Key Laboratory of Genomic and Precision Medicine, Beijing Institute of Genomics, Chinese Academy of Sciences and China National Center for Bioinformation, Beijing 100101, China; University of Chinese Academy of Sciences, Beijing 100049, China; University of Chinese Academy of Sciences, Beijing 100049, China; Aging Biomarker Consortium, Beijing 100101, China; Key Laboratory of Organ Regeneration and Reconstruction, Institute of Zoology, Chinese Academy of Sciences, Beijing 100101, China; Institute for Stem Cell and Regeneration, Chinese Academy of Sciences, Beijing 100101, China; Beijing Institute for Stem Cell and Regenerative Medicine, Beijing 100101, China; State Key Laboratory of Stem Cell and Reproductive Biology, Institute of Zoology, Chinese Academy of Sciences, Beijing 100101, China; Division of Orthopaedics, Quzhou Affiliated Hospital of Wenzhou Medical University, Quzhou 324000, China; CAS Key Laboratory of Genomic and Precision Medicine, Beijing Institute of Genomics, Chinese Academy of Sciences and China National Center for Bioinformation, Beijing 100101, China; University of Chinese Academy of Sciences, Beijing 100049, China; National Genomics Data Center, Beijing Institute of Genomics, Chinese Academy of Sciences and China National Center for Bioinformation, Beijing 100101, China; University of Chinese Academy of Sciences, Beijing 100049, China; Advanced Innovation Center for Human Brain Protection, and National Clinical Research Center for Geriatric Disorders, Xuanwu Hospital Capital Medical University, Beijing 100053, China; Aging Translational Medicine Center, International Center for Aging and Cancer, Xuanwu Hospital, Capital Medical University, Beijing 100053, China; Aging Biomarker Consortium, Beijing 100101, China; State Key Laboratory of Membrane Biology, Institute of Zoology, Chinese Academy of Sciences, Beijing 100101, China; Key Laboratory of Organ Regeneration and Reconstruction, Institute of Zoology, Chinese Academy of Sciences, Beijing 100101, China; Institute for Stem Cell and Regeneration, Chinese Academy of Sciences, Beijing 100101, China; Beijing Institute for Stem Cell and Regenerative Medicine, Beijing 100101, China; CAS Key Laboratory of Genomic and Precision Medicine, Beijing Institute of Genomics, Chinese Academy of Sciences and China National Center for Bioinformation, Beijing 100101, China; University of Chinese Academy of Sciences, Beijing 100049, China; Aging Biomarker Consortium, Beijing 100101, China; Institute for Stem Cell and Regeneration, Chinese Academy of Sciences, Beijing 100101, China

**Keywords:** DNA methylation, aging clock, aging, age prediction

## Abstract

Epigenetic clocks are accurate predictors of human chronological age based on the analysis of DNA methylation (DNAm) at specific CpG sites. However, a systematic comparison between DNA methylation data and other omics datasets has not yet been performed. Moreover, available DNAm age predictors are based on datasets with limited ethnic representation. To address these knowledge gaps, we generated and analyzed DNA methylation datasets from two independent Chinese cohorts, revealing age-related DNAm changes. Additionally, a DNA methylation aging clock (iCAS-DNAmAge) and a group of DNAm-based multi-modal clocks for Chinese individuals were developed, with most of them demonstrating strong predictive capabilities for chronological age. The clocks were further employed to predict factors influencing aging rates. The DNAm aging clock, derived from multi-modal aging features (compositeAge-DNAmAge), exhibited a close association with multi-omics changes, lifestyles, and disease status, underscoring its robust potential for precise biological age assessment. Our findings offer novel insights into the regulatory mechanism of age-related DNAm changes and extend the application of the DNAm clock for measuring biological age and aging pace, providing the basis for evaluating aging intervention strategies.

## Introduction

Aging is a heterogeneous process both within the organism and among individuals, manifested as a gradual decline in tissue and organ function (Cai et al., 2022; López-Otín et al., 2023; Ma et al., 2023a). To precisely estimate one’s biological age (i.e., the level of biological functioning of an individual), which is not identical to the chronological age (i.e., the amount of time since an individual was born), the definition of the aging clock was proposed. Aging clocks are usually built with machine-learning methods that aggregate subsets of important biological features indicative of health status (e.g., clinical measurements, transcripts, proteins) (Field et al., 2018; Rutledge et al., 2022). Among various aging clocks reported to date, the epigenetic clock, which was established based on DNA methylation, is a promising molecular estimator in age measurement (Bao et al., 2023; Horvath and Raj, 2018a; Zhang et al., 2023b; Seale et al., 2022). The DNAm clock has been applied to predict age based on data sourced from several tissues, including whole blood, saliva, brain, liver, etc. (Hannum et al., 2013; Horvath, 2013; Horvath et al., 2018b; Knight et al., 2016). Recent studies indicate that DNA methylation clocks hold promise in reflecting an individual’s health status, disease history, and even mortality (Belsky et al., 2022; Elliott et al., 2021; Levine et al., 2018; McCrory et al., 2021). However, further investigations that can help us better understand the biological implication of age-related DNAm sites and sites constituting the DNAm clock remain crucial.

To date, almost all available DNAm clocks are established in European and American countries. Even so, these clocks still differ in various cohorts (Chen et al., 2016; Horvath et al., 2016; Jansen et al., 2021), leaving it uncertain whether existing DNAm clocks, in spite of high prediction accuracy, would accurately capture aging in cohorts that are not ethnically close to the tested individuals. Indeed, in comparative studies, differences in DNAm alteration across ethnicities have been reported (Adkins et al., 2011; Xia et al., 2014). In addition, reported aging clocks built on different omics datasets, such as transcriptomics, proteomics, and metabolomics, might indeed delineate different aging patterns within an organism (Deelen et al., 2019; Holzscheck et al., 2021; Nie et al., 2022; Williams et al., 2019). Therefore, to comprehensively compare aging multi-omics datasets and potentially integrate DNAm datasets from different ethnic cohorts, we need a deeper understanding of how aging is associated with DNAm (epigenome), other “omes,” and ethnicity.

In this study, we profiled whole blood DNA methylation in a Chinese cohort of individuals of different ages (Quzhou cohort). We also leveraged an independent larger cohort (CAS cohort) to review and validate our findings. In a detailed analysis, we unveiled age-related DNAm alterations and DNAm perturbation hot spots associated with aging. We further built a Chinese DNAm clock, adding a new indicator to the index of Chinese Aging Score (iCAS), named iCAS-DNAmAge, which outperformed several reported DNAm clocks for Chinese individuals. More importantly, we here establish the first DNAm predictors for multi-modal aging clocks. These predicted ages are strongly associated with health status and disease history. Together, this study deepens our understanding of the aging epigenome and provides new tools to estimate the biological age, which might facilitate developing therapeutic strategies for aging and aging-related diseases.

## Results

### Characterization of the age-related DNA methylation changes

**Figure 1.**
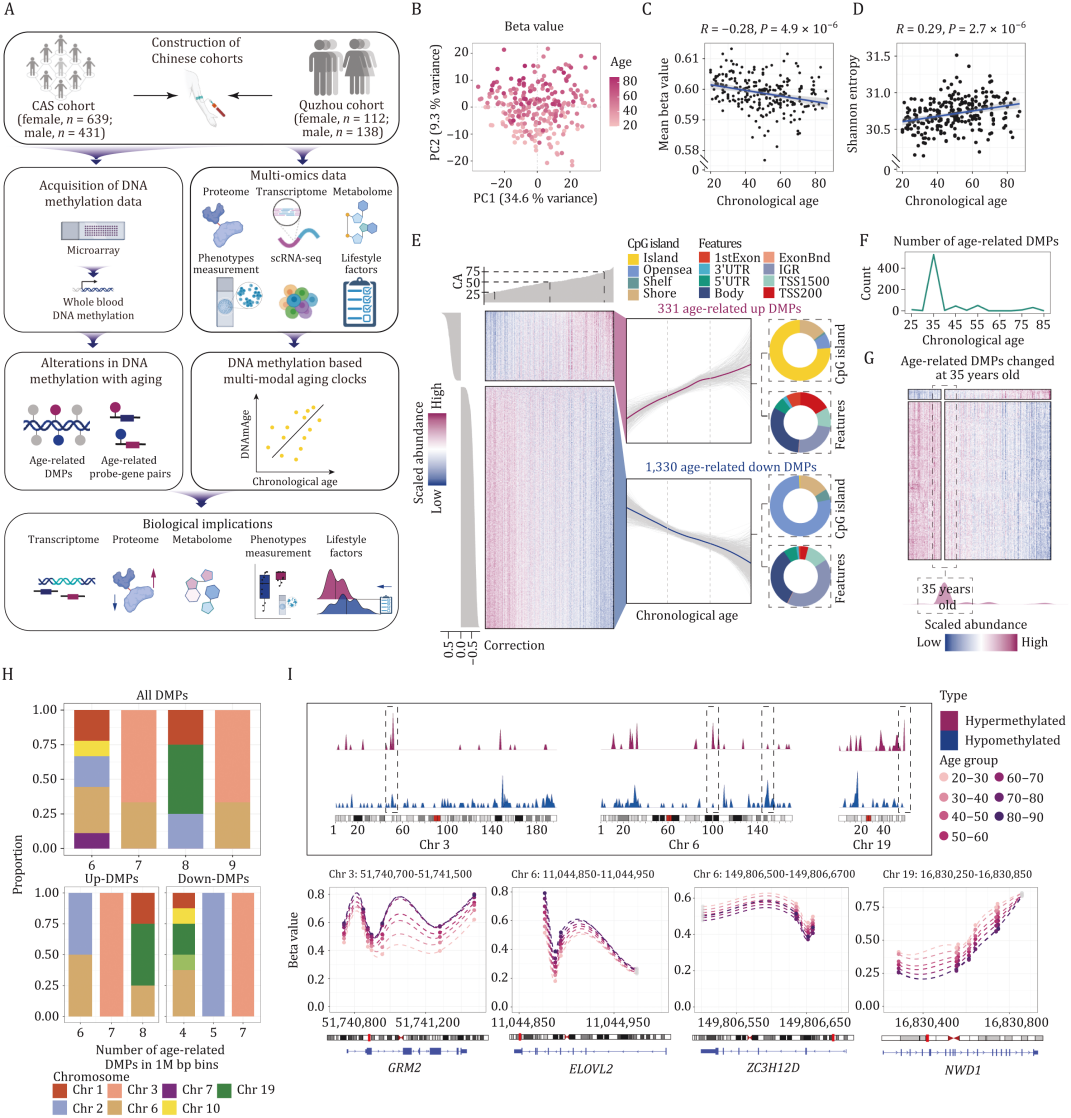
**Methylome analysis reveals age-related changes in methylation levels.** (A) Schematic diagram of this study, involved the identification of age-related DNA methylation characteristics and establishement of the multi-modal DNAm clocks based on the Quzhou and CAS cohorts. The icons were obtained from BioRender.com. (B) Dot plot showing the PCA distribution of methylation patterns. The different shades of color represent different ages. (C) Scatter plot showing the correlation of mean methylation levels with chronological age. Correlation coefficient and *P*-value (Pearson’s correlation analysis) are shown. (D) Scatter plot showing the correlation of Shannon entropy with chronological age. Correlation coefficient and *P*-value (Pearson’s correlation analysis) are shown. (E) Changes of age-related differential methylation positions with age (linear correlation, |ΔBeta/year| > 0.002 and Benjamini-Hochberg adjust *P*-value < 0.01, corrected with BMI, gender and proportion of blood cells). Each row of the heatmap represents a methylation site, and each column represents an individual. The class of the methylation site is annotated. Ring diagram showing the localization of differentially methylated sites (see Methods). (F) Sliding window analysis demonstrating the number of differentially methylated sites that change at different ages. Line plot showing the number of DMPs at different ages. (G) Heatmaps showing the values of features changing at 35 years. (H) Histogram showing the proportion of chromosomes in regions with high age-related methylation variation. (I) Density plot showing the distribution of significantly changing methylation sites on chromosomes with age. LOESS fitting plots showing the average methylation change in the distribution region of differentially methylated hotspots, methylation sites that change linearly with age are colored and the remaining sites are gray (linear correlation, |ΔBeta/year| > 0.002 and Benjamini-Hochberg adjust *P*-value < 0.01, corrected with BMI, gender and proportion of blood cells), and the dashed line showing the average methylation beta value (bottom).

To establish an epigenetic clock that would enable investigations of underlying associations with multi-omics data, we collected whole blood DNA methylation data from a group of individuals aged between 20 and 87 years old (Quzhou cohort from southern China, 112 women, 138 men, Table S1), and subjected biological samples from this cohort to phenomics, transcriptomics, proteomics, metabolomics, and facial image analysis (Li et al., 2023a) (Fig. 1A). In addition, we used a larger cohort (CAS cohort from northern China, 639 women, 431 men) as a validation of the established DNA methylation clock (Du et al., 2019) (Fig. 1A). This comprehensive dataset allowed us to thoroughly profile age-related DNA methylation alterations and build epigenetic predictions into multi-modal aging clocks in the Chinese population.

In the initial principal component analysis (PCA), we identified a significant association between age and DNA methylation (Fig. 1B). We also observed a significant reduction in global DNA methylation level along with an elevation in entropy of methylation in the elderly of both genders (Fig. 1C and 1D). Taken together, these results suggest aging is associated with dramatic changes in DNA methylation. Then, we calculated the age-related differential methylation positions (DMPs) (change in beta per year of age > 0.002 and BH-adjusted *P*-value < 0.05, corrected with blood cell proportions, gender, and BMI values) to identify 331 and 1,330 DMPs that were upregulated and downregulated with age, respectively (Fig. 1E; Table S2). After annotating these age-related DMPs, we observed that DMPs upregulated with age were primarily located within CpG island regions, whereas DMPs downregulated with age were predominantly isolated CpGs situated outside any CpG island, falling into the open sea region (Fig. 1E). Additionally, the percentage of hypermethylated sites situated within 200 bp upstream of the transcription start site (TSS) reached 16% (Fig. 1E). These findings indicate that hypermethylated sites may be more likely to regulate gene expression during aging (Marttila et al., 2015; Pal and Taylor, 2016). Moreover, pathway enrichment analysis suggested that DMPs upregulated with age are involved in various biological processes, particularly pathways associated with development, as identified in previous research (Lu et al., 2023a), while those downregulated with age are enriched for cell morphogenesis (Fig. S1C).

Of note, we observed a comparatively higher concentration of DMPs in the TSS regions of chromosomes 3, 6, and 19 compared to other chromosomes (Fig. 1H and 1I). Within chromosome 3, the hypermethylated sites were primarily situated in the regions of the *GRM2* gene (Fig. 1I), whose methylation level has been reported positive associated with age (Naue et al., 2017). In chromosome 6, the hypomethylated sites were predominantly located in the regions of the *ZC3H12D* gene (Fig. 1I), which encodes MCPIP4, a CCCH-zinc finger family protein known to regulate the pro-inflammatory activation of macrophages (Liang et al., 2008). The hypermethylated sites of chromosome 6 were mainly found in the gene segments of *ELOVL2,* which has been strongly associated with aging in various tissues (Garagnani et al., 2012a; Slieker et al., 2018). In chromosome 19, the methylation level of NACHT and WD repeat domain containing 1 (*NWD1*) was observed to be lower in the elderly (Fig. 1I), and its DNA methylation levels have previously been suggested as a marker for aging (Chen et al., 2022). Collectively, these findings highlight the genomic sites on chromosomes 3, 6, and 19 as hotspots for age-related DNA methylation changes.

In addition to linear change with age, we also tried to identify crests of DNA methylation alterations across age stages. In sliding window analysis, we found that the number of DMPs peaked at the age of 35 in both genders (Fig. 1F; Table S3). Furthermore, women had an additional peak of methylation changes at age 55 that was not observed in men (Fig. S2F). Although the number of DMPs at 55 is nearly twice the number of DMPs at 35 in women, the number of hypomethylated DMPs was high in both age stages (Fig. S2G), suggesting that loss of DNA methylation might occur at earlier stages of lifespan (Figs. 1G, S2F, S2G, S3F, and S3G).

### Integrative analysis of the aging epigenome and transcriptome

**Figure 2.**
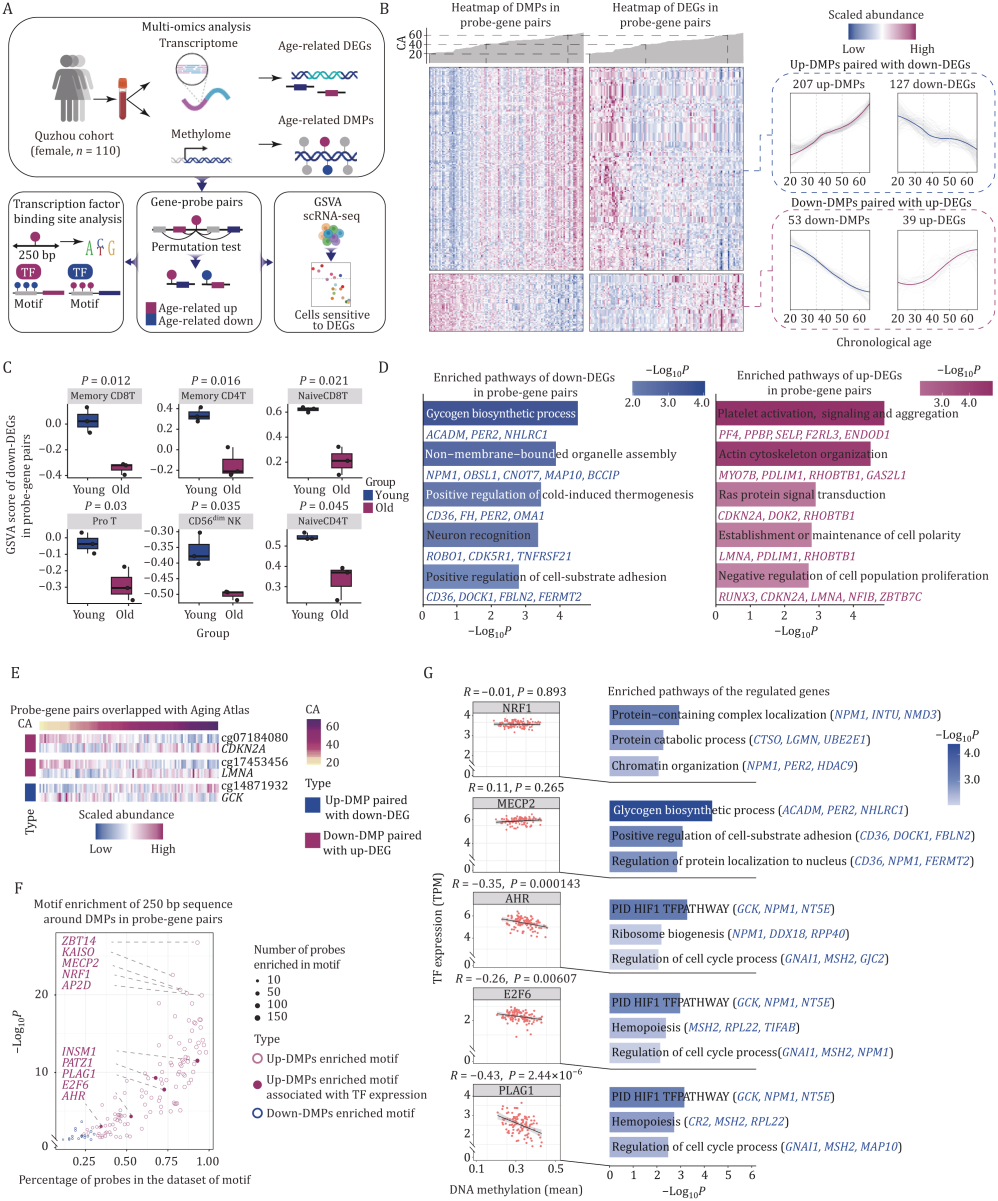
**Integrative analysis of methylome and transcriptome reveals potential DNAm-dependent transcription programs altered by aging.** (A) Schematic diagram showing the process of probe-gene pairs obtaining. TF binding sites are calculated with sequences around the probes, and the extent is calculated to which this part of the gene is altered during aging in single-cell RNA-seq. (B) Heatmaps and line plots demonstrating the presence of potential regulatory relationship sets with age-upregulated methylation-downregulated differential gene pairs and age-downregulated methylation-upregulated differential gene pairs (empirical *P*-value < 0.05). Each row represents a gene/methylation site, each column represents an individual. CA: chronological age. (C) Box plots showing differences in differential genes GSVA scores in single-cell RNA-seq between young and old individuals. (D) Bar plots showing pathways of differential gene enrichment. Down-regulated differential genes enriched pathways are shown in blue (left), and up-regulated differential genes enriched pathways are shown in red (right). (E) The variation of differential genes in the Aging Atlas database with age. Rows of the heatmap represent the gene, and each column represents the individual. CA: chronological age. (F) Dot plot showing results of transcription factor binding site motif analysis of differentially methylated sites associated with transcription (lower OR > 1.1 and Benjamini-Hochberg adjust *P*-value < 0.05). (G) The correlations of transcriptional expression levels of transcription factors and the methylation level of the TF motif binding site. Left, scatter plots showing the correlation between the mean methylation values of the motif and the expression of TF binding to the motif. Right, bar plots showing the enriched pathways of age-decreased genes potentially regulated by TFs.

To interpret the biological implication of the aging epigenome, we performed an integrative analysis of DNA methylation and both bulk and single-cell transcriptomic data from the same cohort. This strategy allowed us to identify age-related probe-gene pairs (Yao et al., 2015), including key transcription factors (TFs) potentially whose activities regulated by DNA methylation, and estimate how these genes change with age in a cell type-specific manner (Fig. 2A). In total, we obtained 207 age-upregulated DMPs with downregulated nearby genes (127 in total), and 53 age-downregulated DMPs with upregulated nearby genes (39 in total) (Fig. 2B; Table S4). In gene set variation analysis (GSVA) based on the scRNA-seq data, we demonstrate that these 166 genes were preferentially changed in T-cell subsets (e.g., Naïve/Memory CD8T and Naïve CD4T) (Fig. 2C; Table S5), suggesting that the DNA methylation state of T cell populations is sensitive to aging. By comparing these genes with genes documented in the Aging Altas (Aging Altas, 2021) we found that several known aging-related genes were potentially regulated by the altered DNA methylation (Fig. 2E). For example, *CDKN2A*, a canonical marker for cellular aging (Krishnamurthy et al., 2004), is upregulated during aging and hypomethylated in the elderly (Fig. 2E), consistent with the results recorded in the Lineage Landscape (Yan et al., 2023). Furthermore, the activated probe-gene pairs (decreased DNA methylation and increased expression) were enriched in pathways such as platelet activation, Ras protein signal transduction, while the repressed probe-gene pairs (increased DNA methylation and decreased expression) are associated with glycogen biosynthetic process and positive regulation of cold-induced thermogenesis, both of which were closely related to homeostasis (Ngo and Steyn, 2015; Wang et al., 2022) (Fig. 2D).

To predict which TFs might bind to genomic loci with aging DMPs, we examined the overlap between probe-gene pairs identified here and TFs whose recognition sequence motifs were documented in the HOCOMOCO database (Kulakovskiy et al., 2016) (see Methods). The results inferred that a larger number of TFs were enriched on upregulated DMPs than on downregulated DMPs (Fig. 2F; Table S6). TFs predicted to bind upregulated DMPs with the highest enrichment scores included NRF1, ZBT14, KAISO, MECP2, etc. (Fig. 2F). Methyl CpG binding protein 2 (MeCP2) acts as a transcriptional regulator by binding to methylated DNA on CpG islands to repress transcription (Meehan et al., 1992), supporting that our analysis can identify potential TFs contributing to the regulatory network of the aging methylome. Consistently, loss of nuclear respiratory factor 1 (NRF1), a key regulator in proteostasis maintenance (Cui et al., 2021; Lehrbach and Ruvkun, 2019), is thought to contribute to aging especially in naïve T cells, causing loss of chromatin accessibility at gene promoters (Moskowitz et al., 2017a, 2017b). We also noticed that several repressed genes in the identified probe-gene pairs were NRF1 target genes, and associated with proteostasis, including *NPM1*, *NMD3*, *USP444* (Matsuura-Suzuki et al., 2022; Thakur et al., 2015; Zhang et al., 2012) (Figs. 2G and S1E). In all, these results indicate that NRF1 regulates the loss of proteostasis in a DNA methylation-dependent manner.

We also screened for TFs for which expression levels were also changed with aging, and in an inverse direction with DNA methylation level of their binding sites. We only identified five TFs that met this criterion: AHR, E2F6, INSM1, PATZ1, and PLAG1 (Figs. 2G and S1G). Interestingly, the aryl hydrocarbon receptor (AHR) was identified as a stress-induced DNA methylation reader (Habano et al., 2022) associated with the CD8T cells activation (Zhang et al., 2023c). Additionally, E2F6 and ZBT14 co-regulate transcriptional expression with AHR, and are also enriched in the hypermethylated DMPs (Oshchepkova et al., 2020; Puga et al., 2000). These results imply that AHR and its co-regulators play a key role in age-related methylation changes. Taken together, our findings establish a DNA methylation-dependent TF regulatory network that is associated with various aging-related pathways and provides insights into the epigenetics mechanism of immune cell aging.

### Establishment of the DNA methylation clock

**Figure 3.**
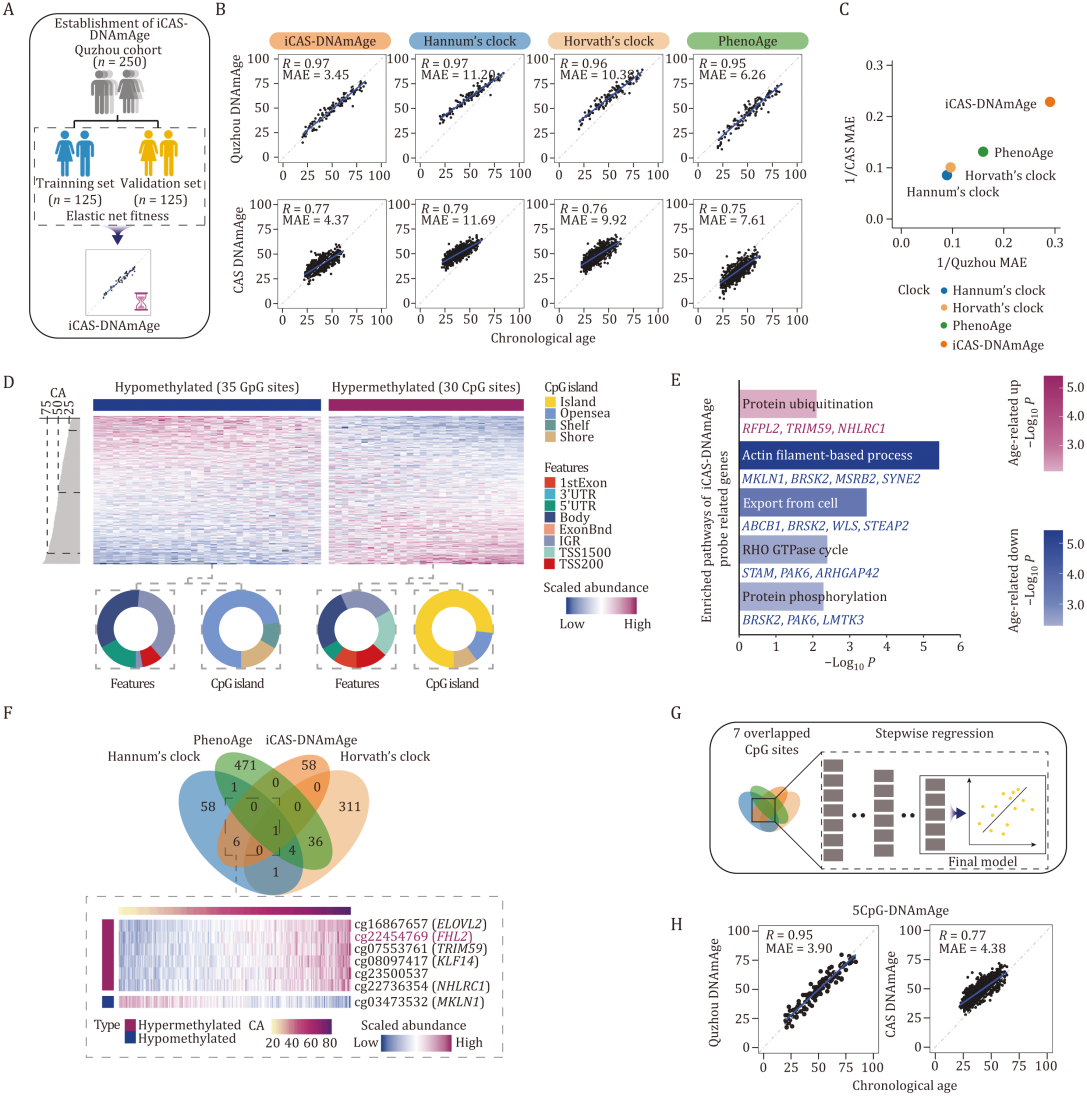
**Establishment of the DNA methylation aging clock for Chinese cohorts.** (A) Schematic diagram showing the process of establishing DNA methylation clock. The individuals in Quzhou cohort (*n* = 250) were randomly divided into a training set (*n* = 125) and a validation set (*n* = 125), and the elastic network was used to construct a DNA methylation clock. (B) Scatter plots of the predicted age of the methylation clock and its linear relationship with age, showing the predicted age of iCAS-DNAm for validation set (*n* = 56), and the predicted age of all individuals (*n* = 112) for other clocks in the Quzhou cohort. The methylation clock-predicted ages for the healthy individuals (*n* = 689) of the CAS cohort are shown at the bottom. Correlation coefficients (Pearson’s correlation analysis) and MAE are shown. (C) Predictive performance of clocks, in which dot plots show the predictive performance of different clocks in the Quzhou and CAS cohorts, the x-axis corresponds to the Quzhou cohort, and the y-axis corresponds to the CAS cohort. MAE: mean absolute error. (D) CpG sites used to predict iCAS-DNAm age. Top panel, circular graph showing the positional information of these CpG sites, with different colors representing different types of positions, and below, heatmap showing the change in clock use of CpG sites with age, each column representing a gene, each row representing an individual. CA: chronological age. (E) Bar plots showing enriched pathways of probe-related genes. Down-regulated differential genes enriched pathways are shown in blue, and up-regulated differential genes enriched pathways are shown in red. (F) The intersection of different clocks using features. Venn plot showing the overlap of different clocks. Heatmap showing the overlap methylation sites of iCAS-DNAmAge and other clocks changing in Quzhou cohort. CA: chronological age. (G) Schematic diagram showing the process of establishing the 5CpG DNA methylation clock. Seven CpG sites coexisting with other clocks were first selected for linear regression fitting, then stepwise regression was performed to remove sites with low contributions, and methylation clocks fitted with five CpG sites were obtained. (H) Dot plot showing the correlation (Pearson’s correlation analysis) and mean absolute error (MAE) between clock predicted age and chronological age, with Quzhou cohort (left) and CAS cohort (right).

We then built a DNA methylation clock (iCAS-DNAmAge) based on the Quzhou cohort and applied the model performance to healthy individuals of the CAS cohort to validate its robustness (see Methods) (Fig. 3A; Table S7). The iCAS-DNAmAge comprises 65 CpG sites, with 35 being upregulated with age and associated with protein ubiquitination, a pathway closely related to protein homeostasis that is critical in the aging process (Huang et al., 2023; Zhang et al., 2022), while the remaining 30 are downregulated with aging and linked to protein phosphorylation (Fig. 3D and 3E). The results showed that prediction accuracy is high in both the Quzhou and CAS cohorts (*R*_Quzhou_ = 0.97, MAE_Quzhou_ = 3.45, *R*_CAS_ = 0.77, MAE_CAS_ = 4.37) (Fig. 3B). To test the generalization ability of the model, we also compared our model to previous DNA methylation clocks (Hannum et al., 2013; Horvath, 2013; Levine et al., 2018). We were encouraged to find that the performance of the iCAS-DNAmAge model surpassed those of the three previous clocks in both Quzhou (*R*_Hannum’s clock_ = 0.97, MAE_Hannum’s clock_ = 11.20; *R*_Horvath’s clock_ = 0.96, MAE_Horvath’s clock_ = 10.38; *R*_PhenoAge_ = 0.95, MAE_PhenoAge_ = 6.26) and CAS cohort (*R*_Hannum’s clock_ = 0.79, MAE_Hannum’s clock_ = 11.69; *R*_Horvath’s clock_ = 0.76, MAE_Horvath’s clock_ = 9.92; *R*_PhenoAge_ = 0.75, MAE_PhenoAge_ = 7.61) (Fig. 3B and 3C; Tables S8 and S9). This result demonstrates that the iCAS-DNAmAge model is generalized in different Chinese cohorts and suggests that, although the DNAm clock can usually predict age accurately, ethnicity might also affect its age predictions.

To gain mechanism insights into the iCAS-DNAmAge clock, we compared the components of all four models and only found seven probes shared between the iCAS-DNAmAge and previous clocks (Fig. 3F). The methylation levels of probes located at *ELOVL2*, *FHL2*, *KLF14*, *NHLRC1*, and *TRIM59* increase with age, while that at *MKLN1* decreases with age (Fig. 3F). The CpG sites located at *FHL2* appeared in all four clocks above, and their DNA methylation levels have been frequently reported to be associated with aging (Bacalini et al., 2017; Garagnani et al., 2012b; Jung et al., 2019), indicating their close relationship with the aging process. Interestingly, a recent study reported that the combination of methylation levels of *ELOVL2*, *FHL2*, *KLF14*, *TRIM59,* and *C1orf132*/*MIR29B2C* is sufficient for age prediction (Jung et al., 2019). Based on these findings, we picked the 7 CpG sites incorporated in iCAS-DNAmAge and the previous clock, developed a concise DNAm clock with a stepwise regression strategy (Fig. 3G). Ultimately, by using only five CpG sites, including cg16867657 (*ELOVL2*), cg22454769 (*FHL2*), cg08097417 (*KLF14*) (Table S7), we obtained an age prediction model (5CpG-DNAmAge) that achieved an accurate prediction in both the validation set of Quzhou cohort (*R* = 0.95, MAE = 3.90) and the CAS cohort (*R* = 0.82, MAE = 5.37) (Fig. 3H; Table S10).

Furthermore, the genes related to the CpG sites underlying iCAS-DNAmAge were predominantly enriched in export from the cell, RHO GTPase cycle, and protein phosphorylation pathways (Fig. 3E). Among them, RHO GTPase has been shown to impact the proliferation and differentiation of hemocytes and is strongly associated with the senescence of human lymphoblastoid cell lines (Florian et al., 2017; Mulloy et al., 2010). Additionally, protein phosphorylation has been found to affect blood cell morphology and cell adhesion (D’Alessandro et al., 2015), implying a close link between iCAS-DNAmAge and the aging process.

### Predicting multi-modal aging clocks from DNA methylation data

**Figure 4.**
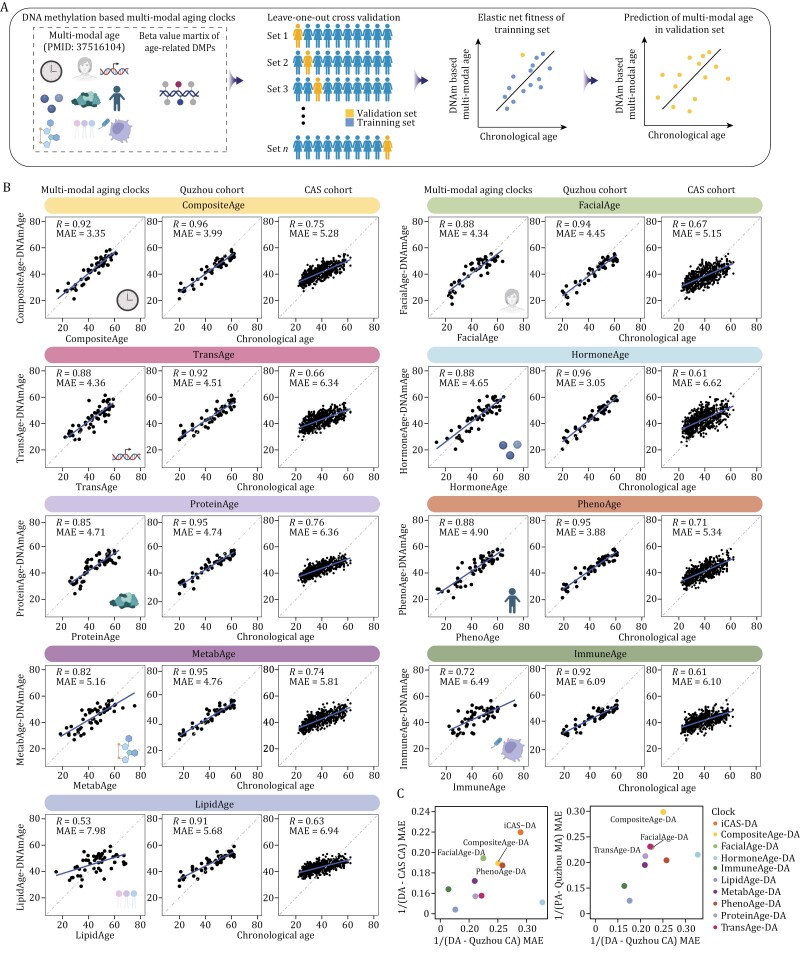
**Establishment of DNA methylation-based predictors for multi-modal aging clocks.** (A) Schematic diagram showing the multi-age methylation clock construction process. (B) Scatter plots showing the predicted age of the age estimators and their linear relationship with age and predicted multi-modal age in Quzhou and CAS cohorts. Correlation coefficients (Pearson’s correlation analysis) and MAE are shown. (C) Dot plots showing the predictive performance of clocks in Quzhou cohort and CAS cohort, with colors indicating different clocks. MAE: mean absolute error; DA: DNAmAge; MA: multi-modal age; CA: chronological age.

Previously, we generated a group of multi-modal aging clocks for female individuals from Quzhou cohort, constructed based on multi-omics and facial image datasets (Li et al., 2023a). Named after their features, these clocks encompass compositeAge, facialAge, transAge, hormoneAge, proteinAge, phenoAge, metabAge, immuneAge, and lipidAge. For instance, compositeAge is derived from the combination of phenomics, transcriptomics, proteomics, and metabolomics datasets, facialAge is generated based on analysis of facial features, and immuneAge is constructed utilizing immune-related features from the transcriptome, proteome, and phenotype. Next, we asked whether these clocks, which reflect aging from both distinct and composite perspectives, could be replaced by the DNA methylation data, which represents an easier method for evaluating biological age by simply estimating the DNA methylation of blood cells (Fig. 4A). Using the leave-one-out cross-validation strategy, we predicted multi-modal ages for female individuals in the validation set (see Methods). Encouragingly, the DNA methylation was proved to be a robust surrogate for these aging clocks. For compositeAge, facialAge, and transAge specifically, which have a high correlation with chronological age, we found that their corresponding DNA methylation clocks were correspondingly more accurate (Fig. 4B and 4C). For example, compositeAge has the best prediction power of chronological age over other clocks based on a single data type, and the prediction results of compositeAge-DNAmAge, also achieve the highest accuracy (*R* = 0.92, MAE = 3.35) (Fig. 4B and 4C). Similarly, facialAge-DNAmAge (*R* = 0.88, MAE = 4.34), hormoneAge-DNAmAge (*R* = 0.88, MAE = 4.65), phenoAge-DNAmAge (*R* = 0.88, MAE = 4.90), transaAge-DNAmAge (*R* = 0.85, MAE = 4.33), proteinAge-DNAmAge (*R* = 0.85, MAE = 4.71), and metabAge-DNAmAge (*R* = 0.82, MAE = 5.13) models exhibit strong correlations with their respective age indices. Though associated with lower accuracy, models built from DNA methylation data can also predict the other clocks, including immuneAge-DNAmAge (*R* = 0.72, MAE = 6.49), lipidAge-DNAmAge (*R* = 0.53, MAE = 7.98) (Fig. 4B and 4C; Table S9).

Subsequently, we applied the DNAm multi-modal clocks to the CAS cohort. We found that the prediction results of compositeAge-DNAmAge and proteinAge-DNAmAge were well correlated with the chronological age (Fig. 4B and 4C; Table S9). The same high correlations were also identified between predicted multi-modal ages and chronological age in the Quzhou cohort (Fig. 4B and 4C). These findings indicate that DNA methylation-based clocks could present a streamlined and consistent method for proxying the complex data provided by multi-omic clocks.

The methylation sites located on *FHL2*, *TRIM59*, *ELVOL2,* and *MKLN1* appeared in multiple methylation clocks, further suggesting a strong correlation between these sites and chronological age (Fig. S1F). Pathway enrichment analysis of clock-specific sites showed that genes related to these sites were enriched in pathways including regulation of Wnt signaling pathway, hematopoiesis, etc., suggesting the DNAm multi-modal clocks may have the ability to reflect different levels of aging (Fig. S1H).

### Paces of DNA methylation-based clocks are associated with various health indicators

**Figure 5.**
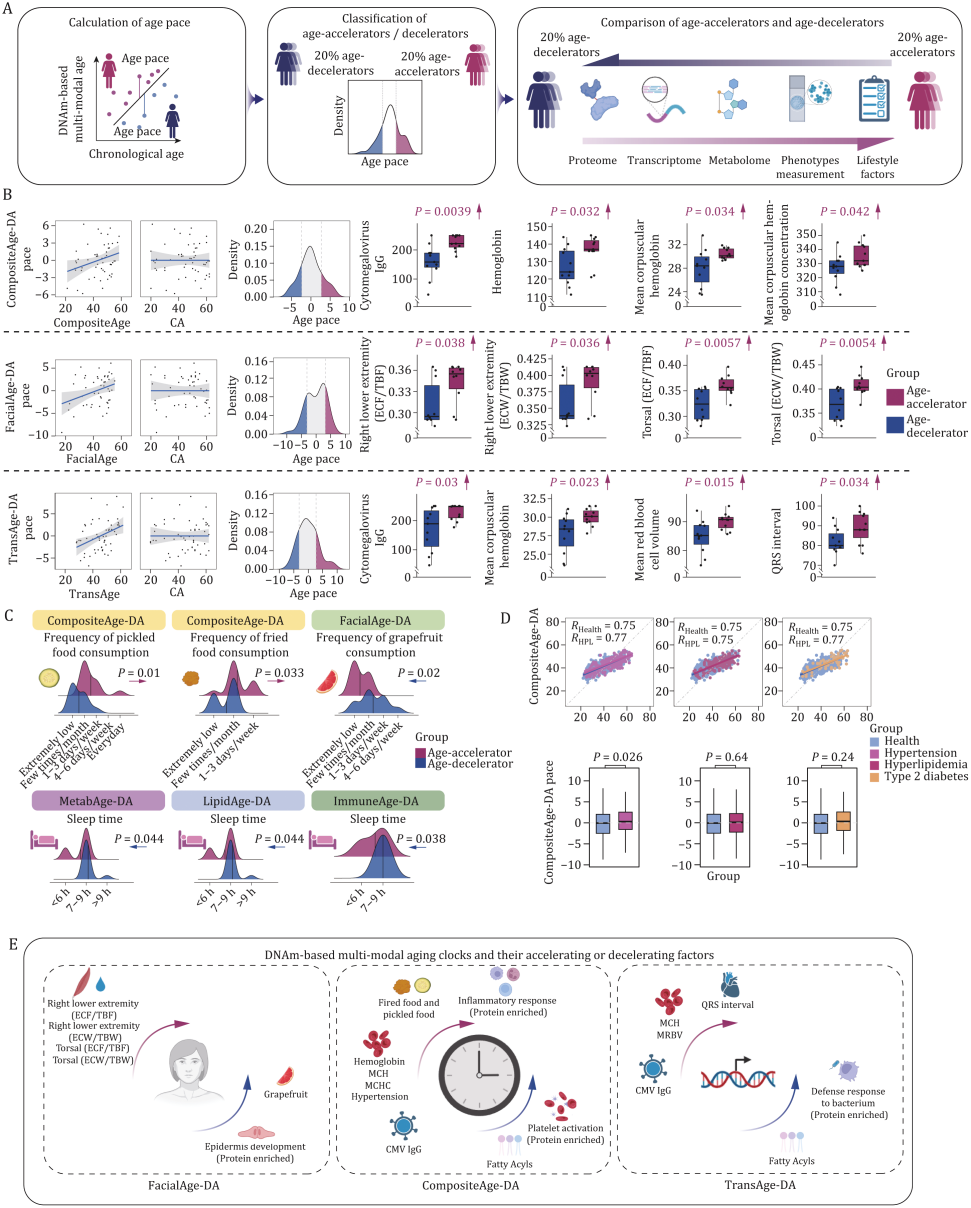
**Association between paces of DNA methylation-based aging clocks and various health indicators.** (A) Schematic diagram showing the calculation of the aging rate. Multi-modal age was used to train the methylation clock and calculate age pace based on chronological age, dividing age-accelerators and age-decelerators based on age pace values. (B) Scatter plots showing the correlation (Pearson’s correlation analysis) between age pace and predicted/chronological age, and density plots showing the division of age-accelerated and age-decelerated groups (left). Box plots showing phenotypic differences between DNAm-based multi-modal age-accelerators and age-decelerators. Each point represents one individual. Right, box plots show the differences between phenotypes and were tested using the *t*-test. ECF/TBF: extracellular fluid/total body fluid; ECW/TBW: extracellular water/total body water; CA: chronological age. (C) Ridge maps showing lifestyle factors of age-accelerators and age-decelerators. DA: DNAmAge. (D) Box plots and scatter plots showing differences in compositeAge-DNAmAge pace between healthy and diseased individuals. DA: DNAmAge. (E) Schematic diagram summarizing the multi-omics changes in DNAm-based multi-modal age accelerators.

Having established the DNAm multi-modal clocks, we postulated that these DNA methylation-based clocks can shed light into what factors contribute to the heterogenous aging process. When we estimated the age pace for different DNAm multi-modal clocks, we did not observe any significant correlation between the age pace and chronological age (Figs. 5B, S5A; Tables S11, S12, S13 and S14), suggesting that individual variations in biological aging were not influenced by age (Elliott et al., 2021). We then identified individuals in the two tails (20%) of the age pace distribution as age-decelerator and age-accelerator. By comparing the multi-omics data, especially the clinical performances of these two groups, we were able to identify key health indicators related to the pace of different aging clocks (Fig. 5A). The results indicated that the compositeAge-DNAmAge exhibited the highest number of multi-omics features associated with its age pace, followed by transAge-DNAmAge and facialAge-DNAmAge (Fig. S6A), suggesting their strong correlation with health status.

Elevated chronological inflammation is an important hallmark of aging (López-Otín et al., 2023; Wu et al., 2024). Here, based on several aging clocks, we found that higher values in age-accelerators were associated with inflammation and immune activation. For example, inflammation-related proteins were upregulated in age-accelerators defined by compositeAge-DNAmAge (CSF1, EXT1, ITGB2), facialAge-DNAmAge (BST1, DPP4), immuneAge-DNAmAge (FCN1, ERAP1), hormoneAge-DNAmAge (IL6R) as well as iCAS-DNAmAge (IL6R, HRNR) (Fig. S6B; Table S12), suggesting the important role of immunosenescence in aging process (Liu et al., 2023; Zhao et al., 2023). Of note, higher cytomegalovirus (CMV) antibody levels, were reported to be associated with various aging-related diseases (Merani et al., 2017; Parry et al., 2016) and accelerated aging in previous studies (Kananen et al., 2015; Poloni et al., 2022; Stowe et al., 2012), was correlated with faster aging based on almost all aging clocks, including compositeAge-DNAmAge, transAge-DNAmAge, metabAge-DNAmAge, and hormoneAge-DNAmAge (Figs. 5B and S6C), implying its instrumental role in affecting the rate of aging. We also found that several proteins down-regulated in the facialAge-DNAmAge accelerated group, including CSTA, KRT2, and TGM3, were associated with epidermal cell differentiation and skin development (Fig. S6B). These results denote how DNAm-based clocks can indicate divergent aging-related molecular changes that link to critical pathological process, such as activated inflammation and impaired immune cell function.

At the phenotypic level, blood-related parameters: hemoglobin (Hb), mean corpuscular hemoglobin (MCH), and mean corpuscular hemoglobin concentration (MCHC) displayed elevation in compositeAge-DNAmAge accelerators (Figs. 5B and S6C). Elevated levels of these parameters have been reported to be associated with chronic hypoxia, like chronic obstructive pulmonary disease and heart disease (Martínez-Quintana and Rodríguez-González, 2013; Woodson et al., 1970). Additionally, transAge-DNAmAge accelerators exhibited elevated MCH and MCHC, along with elongated QRS interval (Figs. 5C and S6B). In facialAge-DNAmAge accelerated group, extracellular fluid/total body fluid (ECF/TBF) and extracellular water/total body water (ECW/TBW) ratios were increased, which were positively correlated with mortality (Kim et al., 2017; Pérez-Morales et al., 2021) (Figs. 5B and S6C). Our analysis revealed that lifestyle choices contributing to an age-accelerating effect were characterized by reduced sleep duration in the context of immuneAge-DNAmAge, lipidAge-DNAmAge, and metabAge-DNAmAge (Fig. 5C). Additionally, for compositeAge-DNAmAge, a diet comprising daily intake of pickled and fried foods was identified (Fig. 5C). Notably, within the extensive CAS cohort, we observed that individuals with hypertension exhibited a significantly higher pace of compositeAge-DNAmAge compared to healthy individuals (Fig. 5D). Taken together, these data demonstrate that DNAm-based clocks can serve as a more convenient alternative to estimate biological age and predict health indicators, and might even be applied to monitor one’s disease status (Fig. 5E).

## Discussion

In this study, we analyzed whole blood DNAm data from Quzhou cohort, revealing key genomic regions, age stages, and potential molecular targets associated with age-related DNAm alterations. We would particularly like to highlight that we here, for the first time, established a Chinese DNAm clock with two independent Chinese cohorts, whose prediction accuracy surpasses those calculated with several other DNAm clock models built in an analysis of cohorts of different ethnicities. These attempts added a new layer for the index of Chinese Aging Score (iCAS) (Li et al., 2023a), and we named it iCAS-DNAm. Furthermore, when we compared a group of multi-modal clocks with our DNAm data, we found that the DNAm-based predictors achieved good accuracy and sensitivity to various health indicators. As a proof of concept, we revealed DNAm as a valuable source to not only predict age but also estimate human biological age from different health aspects. We have uploaded the models to the Human Aging and Longevity Landscape (HALL) database, and the prediction can be easily performed online (Li et al., 2023b).

A previous study suggested that DNAm clocks vary among different cohorts (Horvath et al., 2016). Here, we also noticed that few CpG sites are shared between all clocks, which may be explained by differences in cells, tissues or cohorts of the training set (Bell et al., 2019). Given that China harbors the world’s largest population of the elderly, it is important to build a Chinese cohort-based DNAm clock that accurately evaluates the aging pace of Chinese individuals. The DNAm clock we built here is based on a Chinese cohort of individuals located in southern China, and we validated its generalization performance in another larger independent cohort that consists of volunteers from northern China (Du et al., 2019). Taken together, our data support that our clocks have a good prediction accuracy and outperformed previous DNAm models in the Chinese cohort.

To accurately estimate biological age, only a limited number of CpG sites are necessary (e.g., iCAS-DNAm: 65, Horvath’ clock: 353, Hannum’ clock: 71, PhenoAge: 513) (Hannum et al., 2013; Horvath, 2013; Levine et al., 2018). We also identified a few shared probes among the iCAS-DNAmAge and Hannum clock that have frequently been reported to be associated with aging (Bacalini et al., 2017; Garagnani et al., 2012b; Jung et al., 2019; Park et al., 2016; Wezyk et al., 2018). Among these, fatty acid elongase 2 (*ELOVL2*), a top candidate gene as an aging biomarker based on its methylation levels being highly correlated with age (Garagnani et al., 2012b; Jung et al., 2019), is located on chromosome 6. Here, we observed that age-related DNAm alterations tended to be enriched at chromosome 6. Interestingly, chromosome 6 was defined as an aging hub whose chromatin conformation was closely modulated by *FOXO3*, another top candidate gene associated with longevity (Donlon et al., 2017; Jing et al., 2023; Lei et al., 2021). In future studies, it would be fascinating to unveil the potential biological basis for the high sensitivity of chromosome 6 to aging.

As a dynamic modification associated with responses to both endogenous biological processes and exogenous environmental exposures, DNAm has been used to develop predictors for various health-related applications (Yousefi et al., 2022). These include body mass index (BMI) (Cho et al., 2021), alcohol consumption (Liu et al., 2018), smoking (Bollepalli et al., 2019), neurogenerative disease (Park et al., 2020; Wang et al., 2019), cardiovascular disease (Agha et al., 2019), cancer and cancer prognosis (Luo et al., 2020; Zhang et al., 2017; Zheng et al., 2016). In this study, we built iCAS-DNAmAge and a set of DNAm-based multi-modal aging clocks. These clocks, especially compositeAge-DNAmAge performed well in predicting both age and health status. The acceleration of most DNAm-Age clocks was strongly correlated with the elevation of pro-inflammatory factors and higher cytomegalovirus (CMV) antibody levels. Inflammation has been shown to be closely linked to aging in previous studies (Lu et al., 2023b; Zhang et al., 2023a, d), and CMV has been reported in previous studies as a possible driver of accelerated aging and could affect whole blood DNA methylation levels by influencing hematopoietic ratios (Bergstedt et al., 2022; Lu et al., 2022), and could affect T cell function and phenotype (Hassouneh et al., 2021), which are recognized as characteristics of aging (Li et al., 2022; Lu et al., 2023c). Elevated levels of CMV antibodies are associated with increased CMV infection, and the levels of the former have also been reported to be associated with aging (Waters et al., 2018), indicating the power of DNAmAge in evaluating the aging of the immune system. Moreover, variations in lifestyle parameters, such as less sleep, and lifestyle choices, such as consuming more pickled and fried food, contributed towards predicting the acceleration of certain DNAm-Age clocks, while disease history, such as hypertension, also increased the compositeAge-DNAmAge pace. Thus, the generation of a DNAm-based clock that is integrated with a multi-omics dataset has expanded the composite toolkit for estimating human aging, ultimately helping implement future precision medicines.

Nevertheless, several limitations of this study should be acknowledged. Firstly, the conclusions are drawn from a relatively small, cross-sectional study cohort. Additionally, both the analysis of the association between multi-modal clocks and aging and the association between methylation and transcriptome were performed based on females. However, differences in hormone regulation, physical condition and gene expression (Jia et al., 2023c; Oliva et al., 2020; Ramos et al., 1998) may lead to gender differences in the multi-modal aging clocks. Altogether, our findings provide new insights into the aging epigenome from a multi-omics view. Importantly, the DNAm-based multi-modal aging clocks built in this study broaden our knowledge of DNAm’s ability to predict biological age. This scheme might potentially aid in monitoring the health of the elderly and inform the development of therapeutic strategies for mitigating aging and aging-related diseases.

## Materials and methods

### Human sample collection

The Quzhou cohort was conducted in Quzhou, Zhejiang Province of China. All volunteers signed a written informed consent. Volunteer information was obtained from a previous study (Li et al., 2023a).

### Preparation and storage of biological sample

A 15 mL whole blood sample was collected from each volunteer after fasting overnight. Two EDTA-covered anticoagulation tubes (for plasma and blood cells) were used for collection and transported immediately to the laboratory and stored at 4°C pending further processing. All blood samples were stored at −80°C for long-term preservation.

### DNA preparation

DNA was obtained with the TIANamp Genomic DNA Kit (Tiangen, DP304-03). The quality of the DNA was determined by checking the OD_260_/OD_280_ ratio in the spectrophotometer and the integrity in agarose gels, which should be between 1.8 and 2.0, and the length of the DNA should be greater than 10 kb.

### Microarray experiment of DNA methylation

Genomic DNA (gDNA) samples were bisulfite converted with the EZ DNA Methylation™ Kit (Zymo Research, USA) and then hybridized to the Infinium Methylation EPIC BeadChip array (Illumina, USA) and the Infinium Methylation EPIC v2.0 BeadChip array (Illumina, USA) according to the manufacturer’s instructions. Cases and controls were randomly assigned to each array. The BeadChip array was run in a single base extension reaction, stained, and imaged on an Illumina iScan.

### Processing the DNA methylation data

DNA methylation data for each sample was first processed with ChAMP (version 2.21.1) (Tian et al., 2017), to perform out-of-band Infinium I intensities background correction, RELIC dye bias correction, inter-array normalization, RCP probe type bias correction, low-quality loci filtering, and imputation with the k-nearest neighbor (KNN) method. The beta value matrix was normalized with the BMIQ method (Marabita et al., 2013), and the batch effect was removed with champ.runCombat function (Johnson et al., 2007).

SNP-related probes, multi-hit probes, and probes on the X and Y chromosomes were then deleted referring to previous studies (Nordlund et al., 2013; Zhou et al., 2017), and only the overlapping probes of the two chips were preserved for the pan-gender analysis. All remaining CpG sites were annotated based on the EPICanno.ilm10b4.hg reference (Hansen, 2017).

The probes on the sex chromosomes were retained for methylation analysis of males and females separately (with the X chromosome for females and both the X and Y chromosomes for males). This process involved using the EPICanno.ilm10b4.hg reference annotated for female CpG sites and the manifest file from the Illumina official website for male CpG sites.

CpG sites were annotated based on their position on the genome and CpG island. In this context, CpG island refers to a short stretch of palindromic DNA with a “CpG” sequence. Additionally, CpG shore represents a region within 2 kb around the CpG island, CpG shelf refers to a region within 2 kb around the CpG shore, and Opensea denotes a region outside the aforementioned regions. Furthermore, TSS 200 denotes the region 200 bp upstream of the transcription start site, while TSS 1500 represents the region 1,500 bp upstream of the transcription start site. Moreover, 5ʹUTR and 3ʹUTR represent the 5ʹ and 3ʹ untranslated sequences, 1stExon denotes the first exon, ExonBnd represents the exonic region, Body signifies the gene body region, and IGR stands for the intergenic region (Hansen, 2017).

The beta value for each methylated site was calculated using the formula [M/(M+U)], where M represents the methylation intensity of the site, and U represents the unmethylated intensity of the site, in the range of 0 to 1.

### Estimation of blood cell proportion based on DNA methylation level

ENmix R package (version 1.36.03) (Kassebaum et al., 2016) was used to estimate the proportions of CD8^+^ T cells, CD4^+^ T cells, NK cells, monocytes, B cells, and neutrophils based on FlowSorted.Blood.EPIC dataset (Salas et al., 2018) with the houseman method (Houseman et al., 2012).

### Identification of age-related DMPs

The lmFit function of the limma R package (version 3.54.2) (Ritchie et al., 2015) was applied to fit linear models for the beta value of each methylation site with age as a continuous variable and the proportion of blood cells and BMI as covariates. The *P*-value was corrected using the Benjamin-Hochberg method. The CpG sites with BH-adjusted *P*-value < 0.01 and |ΔBeta/year| > 0.002 were identified as age-related DMPs.The genomicDensity function from the circlize package (version 0.4.15) (Gu et al., 2014) was used to identify the distribution of DMPs within 1M bp bins.

### Identification of peaks in age-related methylation changes

The DEswan package (version 0.0.0.9001) (Lehallier et al., 2019) was used to conduct sliding window analysis for detecting peaks in age-related methylation changes. Statistics analysis were conducted with a sliding window size of 5 years in parcel of 5 years. Those with BH-adjusted *P*-value < 0.05 were considered as significantly changed methylation sites.

### Integrative analysis of DNA methylation and transcriptomics datasets

#### Identification of probe-gene pairs

The transcriptomics data of the blood cells generated in the Quzhou cohort were obtained from a previous study (Li et al., 2023a). Age-related DMPs falling into distal feature regions or promoter regions were first selected with get.feature.probe function in ELMER R package (version 2.24.1) (Yao et al., 2015) to link distal probes with altered DNA methylation to target aging-related differentially expressed genes (DEGs). For each differentially methylated distal probe, the samples in Quzhou cohort were divided into two groups: group M (the 20% highest methylated samples) and group U (the 20% lowest methylated samples), and then the Mann-Whitney U-test was taken to test whether the expression of 10 closest upstream genes and the 10 closest downstream genes of selected DMPs in group M was greater than or equal to that in group U. For each probe-gene pair, the empirical P-value was then calculated using get.permu function in ELMER R package (version 2.24.1) (Yao et al., 2015) to retain probe-gene pairs with empirical *P*-value < 0.05. We then identified probe-gene pairs as those with significant changes in gene expression with age.

#### Motif enrichment analysis of probe-gene pairs

The get.enriched.motif function of the ELMER R package (version 2.24.1) (Yao et al., 2015) was used to perform motif enrichment analysis of regions around probes ± 250 bp that appeared to be up/downregulated with age in the identified probe-gene pairs, using annotation from HOCOMOCO v11 database (Kulakovskiy et al., 2016). A probe set was considered significantly enriched in a particular motif when meeting the criteria: the 95% confidence interval for the ratio (odds ratio) was greater than 1.1, the motif appeared at least 10 times, and the BH-adjusted *P*-value was < 0.05.

#### Identification of the age-related transcription factors for the enriched motifs

For each enriched motif, the average beta value of methylated sites in the motif was calculated. We then performed Pearson’s correlation analysis between the average beta value and the expression level of the TF-coding gene that was enriched in the motif. Those with *P*-value < 0.05 were determined as the age-related TFs that might be regulated by DNA methylation.

### Gene set variance analysis (GSVA) for the DNAm-related aging-DEGs

Genes in probe-gene pairs were selected to perform GSVA with the GSVA R package (version 1.5.0) (Hänzelmann et al., 2013). GSVA scores for different cell types between aged and young individuals were calculated based on the scRNA-seq datasets generated from the Quzhou cohort (Li et al., 2023a). Differences in GSVA scores between young and aged groups were considered significant when *P*-value < 0.05 (*t*-test).

### DNA methylation age prediction

#### DNAm age calculation with previous methylation clocks

Three previous DNAm clocks that were frequently used to obtain DNAmAge in various studies were applied to the datasets of the two cohorts (Bocklandt et al., 2011; Horvath, 2013; Levine et al., 2018). For the calculation of Horvath DNAm clock, the predicted age was transformed using the following equation:


$$\begin{array}{l} f\left(DNAmAge\right)=\left\{ \begin{array}{l} \;DNAmAge\times \left(20+1\right)+20,\;if\;DNAmAge\geq 0\;e^{DNAmAge+ln\left(20+1\right)}-1,\;if\;DNAmAge<0\; \end{array}\right. \end{array}$$

Some probes required for age prediction were filtered in the processing step due to low quality. The missing values in the methylation matrix were imputed with the mean beta value using the dnaMethyAge R package (Wang et al., 2023).

#### Establishment of iCAS-DNAmAge

To build a DNAm clock for Chinese individuals, the 250 healthy individuals from the Quzhou cohort were divided a stratified random sample of male and female individuals into a training set (125 individuals) and a validation set (125 individuals). The chronological age was transformed using the following function, in accordance with the Horvath DNAm clock (Horvath, 2013):


$$\begin{array}{l} f\left(Age\right)=\left\{ \begin{array}{l} \;\left(\;Age+1\right)/\left(20+1\right)-1,\;if\;Age\geq 20\;ln\left(\left(Age+1\right)/\left(20+1\right)\right),\;\;if\;Age<20\; \end{array}\right. \end{array}$$

The ElasticNet regression model was trained with the training set, using the cv.glmnet function of the glmnet R package (version 4.1.4) (Simon et al., 2011) to set the alpha values from 0.1 to 0.9. Lambda values were determined using 10-fold cross-validation on the training set. Model presenting lowest mean absolute error (MAE) in validation set was selected as final model, which was then subjected to the dataset of the CAS cohort. The model predictions were transformed using the f(DNAmAge) function described above.

#### Establishment of DNAm-based multi-modal aging clocks

The DNAm-based multi-modal aging clocks were built with a similar strategy to build the iCAS-DNAmAge, except that the predicted age was changed from chronological age to the multi-modal age obtained in a previous study (Li et al., 2023a). In addition, the leave-one-out method was used, i.e. only one individual in the validation set and all the rest in the training set, to calculate the predicted age for all individuals in the validation set. The model with the lowest MAE was selected as the final model. For the prediction of CAS cohort, the alpha value of the final model was selected, and all the individuals having multi-modal age information in Quzhou cohort were used as a training set to build the DNAm-based multi-modal age clocks.

#### Establishment of 5CpG-DNAmAge

The 5CpG-DNAmAge was established using the stepwise regression method. Initially, a linear regression model was created by incorporating the overlapping CpG sites in the training set with chronological age. Each CpG site was then systematically removed, and its effect on the model was evaluated. Eventually, variables with minimal contributions were eliminated, resulting in a model that included five key CpG sites. This model was then applied to the test sets of the Quzhou cohort and CAS cohort, and its accuracy was assessed using MAE and Pearson’s correlation.

### Association analysis between age acceleration and multi-omics health indicators

#### Definition of age-accelerators and age-decelerators

The age pace was defined as the residual between the predicted and its linear model regression value with chronological age in female validation set, with an age pace greater than 0 classified as accelerated aging and an age pace lower than 0 classified as decelerated aging for the predicted age. The age-accelerators and age-decelerators were then defined as the top and bottom 20% of residual (combining to 40% of all predictors). For each model, we used pcor.test function from ppcor package (version 1.1) (Kim, 2015) to perform a Pearson’s correlation test and verify the correlation between aging pace and chronological age in female individuals.

#### Phenotypic measurements

The differences in phenotypic measurements between the age-accelerators and age-decelerators were analyzed with the *t*-test with a cutoff of *P*-value < 0.05.

#### Lifestyle factors

The differences in life factors between the age-accelerators and age-decelerators were analyzed with the *t*-test with a cutoff of *P*-value < 0.05. The differences in dichotomous life factors, between the age-accelerators and age-decelerators were analyzed with the chi-squared test with a cutoff of *P*-value < 0.05.

#### Transcripts

The DESeq2 package (version 1.36.0) (Love et al., 2014) was applied to identify DEGs between the age-accelerators and age-decelerators with a cutoff of BH-adjusted *P*-value < 0.05 and | Log_2_foldchange | > 0.5.

#### Proteins

The DEP package (version 1.22.0) (Zhang et al., 2018) was used to analyze the protein intensity data. We first filtered the matrix of age-accelerators and age-decelerators and only kept the proteins that were detected in more than two-thirds of the selected individuals. Background correction and normalization were then performed by variance stabilizing transformation. The missing values were imputed according to the KNN method, and the differentially expressed proteins between the age-accelerators and age-decelerators were identified as those with *P*-value < 0.05 and |Log_2_foldchange| > 0.5.

#### Metabolites

The abundance of metabolites of age-accelerators and age-decelerators was first processed with MetaboAnalystR package (version 4.0) (Chong and Xia, 2018), normalized by dividing the value by the mean of each sample, and then subjected to the LogNorm function. The *t*-test was used to perform comparison analysis, and the differentially expressed metabolites between the age-accelerators and age-decelerators were identified as those with *P*-value < 0.05 and |Log_2_foldchange| > 0.5.

### Enrichment analysis of pathways

The Metascape webtool (Zhou et al., 2019) was used to conduct pathway enrichment analysis for genes and proteins.

### Statistics analysis

Statistical analysis of the comparisons in Figs. 2C, 5C, 5D, S4A, S4B and S6B was performed using the two-tailed *t*-test with the stats R package (version 4.2.2). Statistical analysis of the comparisons in Fig. 5E was performed using the Wilcoxon rank-sum test with the ggpubr R package (version 0.4.0).

## Supplementary data

Supplementary data is available at *Protein & Cell Journal* online. https://doi.org/10.1093/procel/pwae011.

pwae011_suppl_Supplementary_Materials

## Data Availability

DNA methylation raw data of Quzhou cohort can be accessed in the OMIX database under the accession number OMIX005455. DNA methylation raw data of CAS cohort will be accessed with reasonable requests. The multi-omics datasets were obtained from a previous study (Li et al., 2023a).

## Code availability

No specific custom script was used in this study. Codes will be accessed with reasonable requests.
