# Supplementary material for: DNA methylation clocks for estimating biological age in Chinese cohorts
Source: Protein Cell. 2024 Mar 14;15(8):575–93. doi: 10.1093/procel/pwae011 (PMC11259550; doi:10.1093/procel/pwae011)
Supplement: pwae011_suppl_Supplementary_Materials [file pwae011_suppl_supplementary_materials.zip › pwae011_suppl_supplementary_figures_s1-s6.pdf]

Supplemental materials

Figure S1. Age-related changes of DNA methylation.

(A) Volcano plot demonstrating changes in methylation sites. The color keys indicate age-downregulated DMPs (blue) and age-upregulated DMPs (red).
(B) Heatmap showing the results of deconvolution of blood cells with the houseman method. Rows represent the cell and columns represent the individuals. The shade of color indicates the proportion of cells. CA: chronological age.
(C) Bar plot showing the enriched pathways of genes related to age-increased (top, red) and age-decreased (bottom, blue) differential DNA methylation sites. Key DMP-related genes enriched in the indicated terms are listed below. The different shade of colors indicates the $-\log_{10}P$ value from low to high.
(D) Bar plots showing the pathway enrichment results of the changing features at 35 years old and key DMP-related genes enriched in the indicated terms are listed below.

(E) Bar plots showing the enriched pathways of age-decreased genes potentially regulated by NRF1. The shade of color indicates the $-\log_{10}P$ value from low to high.
(F) Heatmap showing the most shared sites between different clocks and the methylation changes with aging. The different shades of color on the top represent different ages. Bins are colored from blue to red to indicate low to high methylation levels. CA: chronological age.
(G) Transcription factors whose transcriptional expression levels trend in the opposite direction to the methylation level of the motif binding site. Left, scatter plots showing the correlation between the mean methylation values of the motif and the expression of TF binding to the motif. Right, bar plots showing the enriched pathways of age-decreased genes potentially regulated by TFs. The shade of color indicates the $-\log_{10}P$ value from low to high.
(H) Bar plot showing the number of probes specific to each clock, with up-regulated methylation sites colored in red and down-regulated methylation sites colored in blue (top). Heatmap showing the enriched pathways of probe-related genes (bottom). The different shade of colors indicates the $-\log_{10}P$ value from low to high.

Figure S2. Characterization of the age-related DNA methylation changes in female.

(A) Dot plot showing the PCA distribution of methylation patterns. The different shades of color represent different ages.
(B) Scatter plot showing the correlation of mean methylation levels with chronological age. Correlation coefficient and P-value (Pearson's correlation analysis) are shown.
(C) Scatter plot showing the correlation of Shannon entropy with chronological age. Correlation coefficient and P-value (Pearson's correlation analysis) are shown.
(D) Venn plot showing the overlap between the changed features at two ages (left, age 35; right, age 55).
(E) Changes of age-related differential methylation positions with age (linear correlation, $|\Delta Beta/year| > 0.002$ and Benjamini-Hochberg adjust P value < 0.01, corrected with BMI, gender and proportion of blood cells). Each row of the heatmap represents a methylation site, and each column represents an individual. Bins are

colored from blue to red to indicate low to high methylation levels. CA: chronological age.
(F) Sliding window analysis demonstrating the number of differentially methylated sites that change at different ages. Line plot showing the number of DMPs at different ages.
(G) Heatmaps showing the scale values of features changing at 35 years old (top) and 55 years old (bottom). The color of the bins indicates the scaled abundance of beta value from low (blue) to high (red).
(H) Bar plots showing the pathway enrichment results of the changing features at 35 and 55 years old and key DMP-related genes enriched in the indicated terms are listed below.

(I) Histogram showing the proportion of chromosomes in regions with high age-related methylation variation. The colors of the pots indicate different chromosomes.

(J) Density plot showing the distribution of significantly changing methylation sites on chromosomes with age, with up-regulated methylation sites colored in red and down-regulated methylation sites colored in blue (top). LOESS fitting plots showing the average methylation change in the distribution region of differentially methylated hotspots, methylation sites that change linearly with age are colored and the remaining sites are grey (linear correlation, $|\Delta\text{Beta/year}| > 0.002$ and Benjamini-Hochberg adjust P-value < 0.01, corrected with BMI and proportion of blood cells), and the dashed line showing the average methylation beta value (bottom).

Figure S3. Characterization of the age-related DNA methylation changes in male.

(A) Dot plot showing the PCA distribution of methylation patterns. The different shades of color represent different ages.
(B) Scatter plot showing the correlation of mean methylation levels with chronological age. Correlation coefficient and P-value (Pearson's correlation analysis) are shown.
(C) Scatter plot showing the correlation of Shannon entropy with chronological age. Correlation coefficient and P-value (Pearson's correlation analysis) are shown.
(D) Venn plot showing the overlap between the changed features at two ages (left, age 35; right, age 80).
(E) Changes of age-related differential methylation positions with age (linear correlation, $|\Delta\text{Beta/year}| > 0.002$ and Benjamini-Hochberg adjust P value < 0.01, corrected with BMI and proportion of blood cells). Each row of the heatmap represents a methylation site, and each column represents an individual. Bins are colored from blue to red to indicate low to high methylation levels. CA: chronological age.
(F) Sliding window analysis demonstrating the number of differentially methylated sites that change at different ages. Line plot showing the number of DMPs at different ages.
(G) Heatmaps showing the scale values of features changing at 35 years old (top) and 55 years old (bottom). The color of the bins indicates the scaled abundance of beta value from low (blue) to high (red).
(H) Histogram showing the proportion of chromosomes in regions with high age-related methylation variation. The colors of the pots indicate different chromosomes.

(I) Density plot showing the distribution of significantly changing methylation sites on chromosomes with age, with up-regulated methylation sites colored in red and down-regulated methylation sites colored in blue (top). LOESS fitting plots showing the average methylation change in the distribution region of differentially methylated hotspots, methylation sites that change linearly with age are colored and the remaining sites are grey (linear correlation, |ΔBeta/year| > 0.002 and Benjamini-Hochberg adjust P-value < 0.01, corrected with BMI, gender and proportion of blood cells), and the dashed line showing the average methylation beta value (bottom).

Figure S4. GSVA of genes in the age-related probe-gene pairs.

(A) GSVA of age-upregulated genes in probe-gene pairs. Box plots show the GSVA scores, with points representing the individual, and color keys indicate the age-accelerators (red) and age-decelerators (blue).
(B) GSVA of age-downregulated genes in probe-gene pairs. Box plots show the GSVA scores, with points representing the individual, and color keys indicate the age-accelerators (red) and age-decelerators (blue).

Figure S5. Age pace of DNA methylation-based multi-model aging clocks.

(A) Relationship between age pace predicted by DNAm-based multi-model age clock and multi-model age/chronological age. Scatter plots showing the correlation (Pearson's correlation analysis) between age pace and predicted age/ chronological age. Density plots of age pace, and the color keys indicate the age-accelerators (red) and age-decelerators (blue).

Figure S6. Associations between age pace of DNA methylation-based multi-model aging clocks and multi-omics features.

(A) Dot plots showing the multi-omics changed features in DNAm-based multi-modal age accelerators. Colors indicate the type of DNAm-based multi-modal age. DA: DNAmAge.
(B)Dot plots showing the number of features associated with age accelerated predicted by different clocks, and the color keys indicate the clock types. Heatmap showing differential genes, metabolites, and proteins, with rows representing the multi-model ages, columns representing the features, and the color keys indicate the differences and the class of features. The shade of color in the heatmap indicates the scaled abundance of features measurement.
(C) Heatmap shows differential phenotypes (T-test, P-value < 0.05), with rows corresponding to DNAm-based multi-model ages, columns corresponding to features, and the classification of the measurement phenotypes is annotated as bar plots above. The shade of color indicates the Log2foldchange value from low to high.

Supplementary Table Legends

Table S1. The distribution of age and gender in cohorts.
Table S2. Age-related differential methylation positions.
Table S3. Sliding window analysis results.
Table S4. Age-related probe-gene pairs.
Table S5. Gene set variance analysis differences between young and old for the DNAm-related aging-DEGs.

Table S6. Motif enrichment of probe-gene pairs.
Table S7. The final models to build the DNAm-based aging clocks.
Table S8. The predicted age of previous DNAm clocks.
Table S9. The predicted age of DNAm-based multi-model age clocks. Table S10. The predicted age of 5 CpGs clock.
Table S11. DNAm-based multi model age pace related metabolites (age-accelerators vs age-decelerators).
Table S12. DNAm-based multi model age pace related proteins (age-accelerators vs age-decelerators).
Table S13. DNAm-based multi model age pace related genes (age-accelerators vs age-decelerators).
Table S14. DNAm-based multi model age pace related phenotypes and life factors (age-accelerators vs age-decelerators).

Fig S1

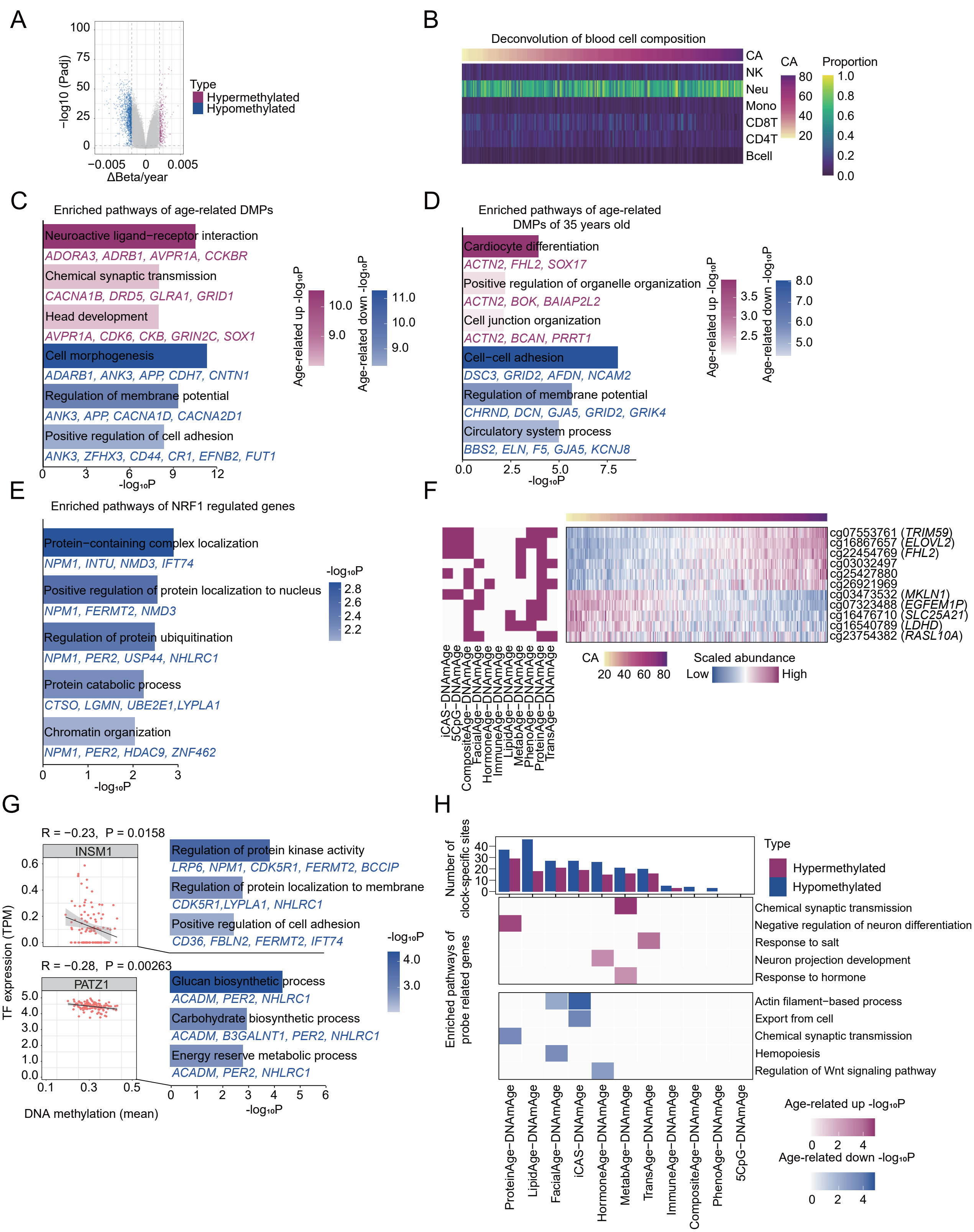
A
−log10 (Padj)
ΔBeta/year
Type
Hypermethylated
Hypomethylated
B
Deconvolution of blood cell composition
CA
NK
Neu
Mono
CD8T
CD4T
Bcell
Proportion
C
Enriched pathways of age-related DMPs
Neuroactive ligand−receptor interaction
ADORA3, ADRB1, AVPR1A, CCKBR
Chemical synaptic transmission
CACNA1B, DRD5, GLRA1, GRID1
Head development
AVPR1A, CDK6, CKB, GRIN2C, SOX1
Cell morphogenesis
ADARB1, ANK3, APP, CDH7, CNTN1
Regulation of membrane potential
ANK3, APP, CACNA1D, CACNA2D1
Positive regulation of cell adhesion
ANK3, ZFHX3, CD44, CR1, EFNB2, FUT1
-log₁₀P
Age-related up -log₁₀P
Age-related down -log₁₀P
D
Enriched pathways of age-related DMPs of 35 years old
Cardiocyte differentiation
ACTN2, FHL2, SOX17
Positive regulation of organelle organization
ACTN2, BOK, BAIAP2L2
Cell junction organization
ACTN2, BCAN, PRRT1
Cell−cell adhesion
DSC3, GRID2, AFDN, NCAM2
Regulation of membrane potential
CHRND, DCN, GJA5, GRID2, GRIK4
Circulatory system process
BBS2, ELN, F5, GJA5, KCNJ8
E
Enriched pathways of NRF1 regulated genes
Protein−containing complex localization
NPM1, INTU, NMD3, IFT74
Positive regulation of protein localization to nucleus
NPM1, FERMT2, NMD3
Regulation of protein ubiquitination
NPM1, PER2, USP44, NHLRC1
Protein catabolic process
CTSO, LGMN, UBE2E1,LYPLA1
Chromatin organization
NPM1, PER2, HDAC9, ZNF462
F
cg07553761 (TRIM59)
cg16867657 (ELOVL2)
cg22454769 (FHL2)
cg03032497
cg25427880
cg26921969
cg03473532 (MKLN1)
cg07323488 (EGFEM1P)
cg16476710 (SLC25A21)
cg16540789 (LDHD)
cg23754382 (RASL10A)
iCAS−DNAmAge
5CpG−DNAmAge
CompositeAge−DNAmAge
FacialAge−DNAmAge
HormoneAge−DNAmAge
ImmuneAge−DNAmAge
LipidAge−DNAmAge
MetabAge−DNAmAge
PhenoAge−DNAmAge
ProteinAge−DNAmAge
TransAge−DNAmAge
Scaled abundance
Low
High
G
R = −0.23, P = 0.0158
INSM1
R = −0.28, P = 0.00263
PATZ1
TF expression (TPM)
DNA methylation (mean)
Regulation of protein kinase activity
LRP6, NPM1, CDK5R1, FERMT2, BCCIP
Regulation of protein localization to membrane
CDK5R1,LYPLA1, NHLRC1
Positive regulation of cell adhesion
CD36, FBLN2, FERMT2, IFT74
Glucan biosynthetic process
ACADM, PER2, NHLRC1
Carbohydrate biosynthetic process
ACADM, B3GALNT1, PER2, NHLRC1
Energy reserve metabolic process
ACADM, PER2, NHLRC1
H
Number of clock-specific sites
Type
Hypermethylated
Hypomethylated
Enriched pathways of probe related genes
Chemical synaptic transmission
Negative regulation of neuron differentiation
Response to salt
Neuron projection development
Response to hormone
Actin filament−based process
Export from cell
Chemical synaptic transmission
Hemopoiesis
Regulation of Wnt signaling pathway
ProteinAge−DNAmAge
LipidAge−DNAmAge
FacialAge−DNAmAge
iCAS−DNAmAge
HormoneAge−DNAmAge
MetabAge−DNAmAge
TransAge−DNAmAge
ImmuneAge−DNAmAge
CompositeAge−DNAmAge
PhenoAge−DNAmAge
5CpG−DNAmAge
Age-related up -log₁₀P
Age-related down -log₁₀P

Fig S2

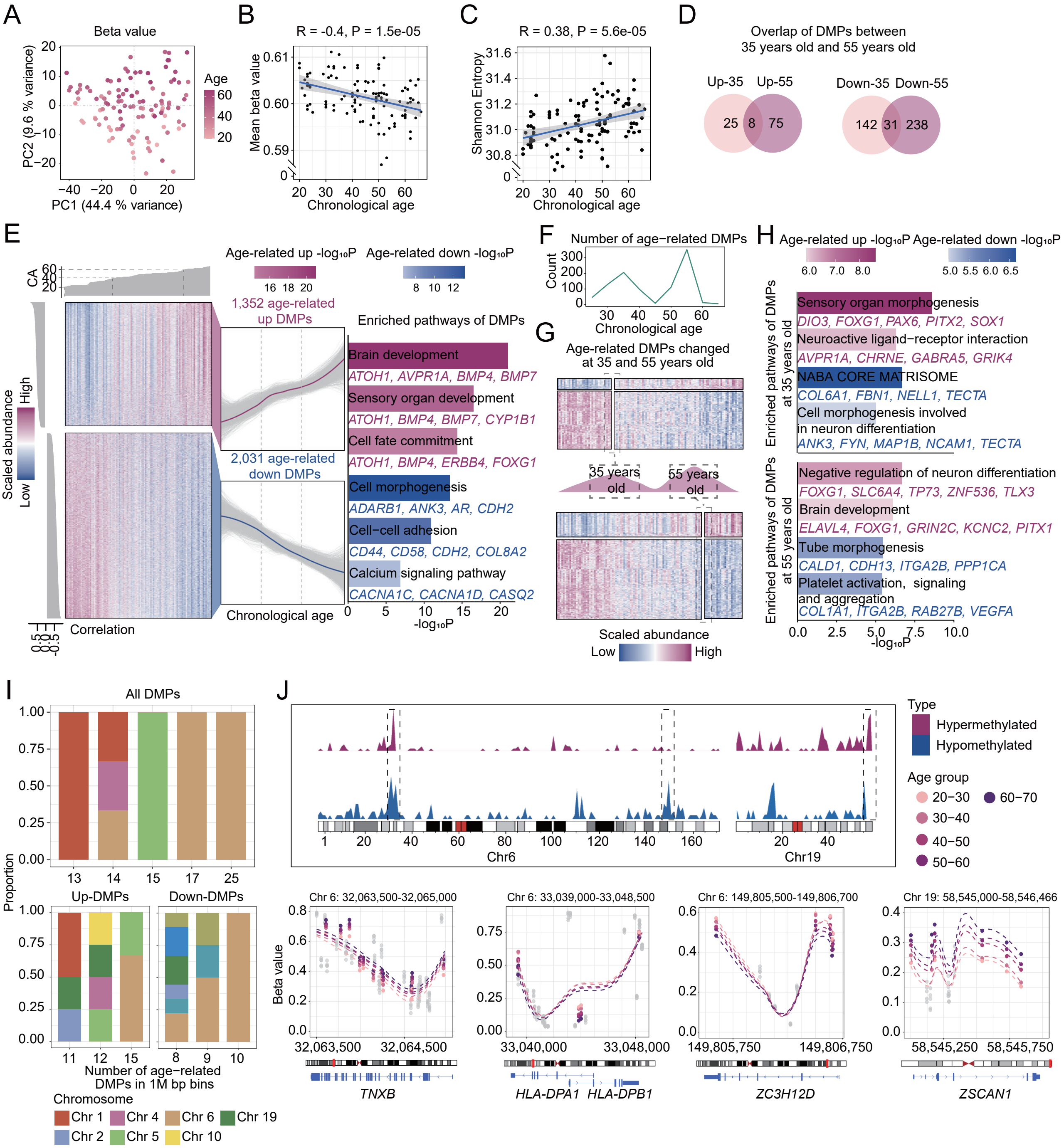
A
Beta value
PC2 (9.6 % variance)
PC1 (44.4 % variance)
Age
B
R = -0.4, P = 1.5e-05
Mean beta value
Chronological age
C
R = 0.38, P = 5.6e-05
Shannon Entropy
Chronological age
D
Overlap of DMPs between
35 years old and 55 years old
Up-35 Up-55
25 8 75
Down-35 Down-55
142 31 238
E
CA
Age-related up -log₁₀P
Age-related down -log₁₀P
1,352 age-related up DMPs
2,031 age-related down DMPs
Enriched pathways of DMPs
Brain development
ATOH1, AVPR1A, BMP4, BMP7
Sensory organ development
ATOH1, BMP4, BMP7, CYP1B1
Cell fate commitment
ATOH1, BMP4, ERBB4, FOXG1
Cell morphogenesis
ADARB1, ANK3, AR, CDH2
Cell−cell adhesion
CD44, CD58, CDH2, COL8A2
Calcium signaling pathway
CACNA1C, CACNA1D, CASQ2
-log₁₀P
Chronological age
Scaled abundance
High
Low
Correlation
F
Number of age−related DMPs
Count
Chronological age
G
Age-related DMPs changed
at 35 and 55 years old
35 years old
55 years old
Scaled abundance
Low High
H
Age-related up -log₁₀P
Age-related down -log₁₀P
Enriched pathways of DMPs at 35 years old
Sensory organ morphogenesis
DIO3, FOXG1, PAX6, PITX2, SOX1
Neuroactive ligand−receptor interaction
AVPR1A, CHRNE, GABRA5, GRIK4
NABA CORE MATRISOME
COL6A1, FBN1, NELL1, TECTA
Cell morphogenesis involved
in neuron differentiation
ANK3, FYN, MAP1B, NCAM1, TECTA
Enriched pathways of DMPs at 55 years old
Negative regulation of neuron differentiation
FOXG1, SLC6A4, TP73, ZNF536, TLX3
Brain development
ELAVL4, FOXG1, GRIN2C, KCNC2, PITX1
Tube morphogenesis
CALD1, CDH13, ITGA2B, PPP1CA
Platelet activation, signaling
and aggregation
COL1A1, ITGA2B, RAB27B, VEGFA
-log₁₀P
I
All DMPs
Proportion
Up-DMPs
Down-DMPs
Number of age−related DMPs in 1M bp bins
Chromosome
Chr 1 Chr 4 Chr 6 Chr 19
Chr 2 Chr 5 Chr 10
J
Type
Hypermethylated
Hypomethylated
Age group
20−30 30−40 40−50 50−60 60−70
Chr6
Chr19
Chr 6: 32,063,500-32,065,000
Chr 6: 33,039,000-33,048,500
Chr 6: 149,805,500-149,806,700
Chr 19: 58,545,000-58,546,466
Beta value
TNXB
HLA-DPA1 HLA-DPB1
ZC3H12D
ZSCAN1

Fig S3

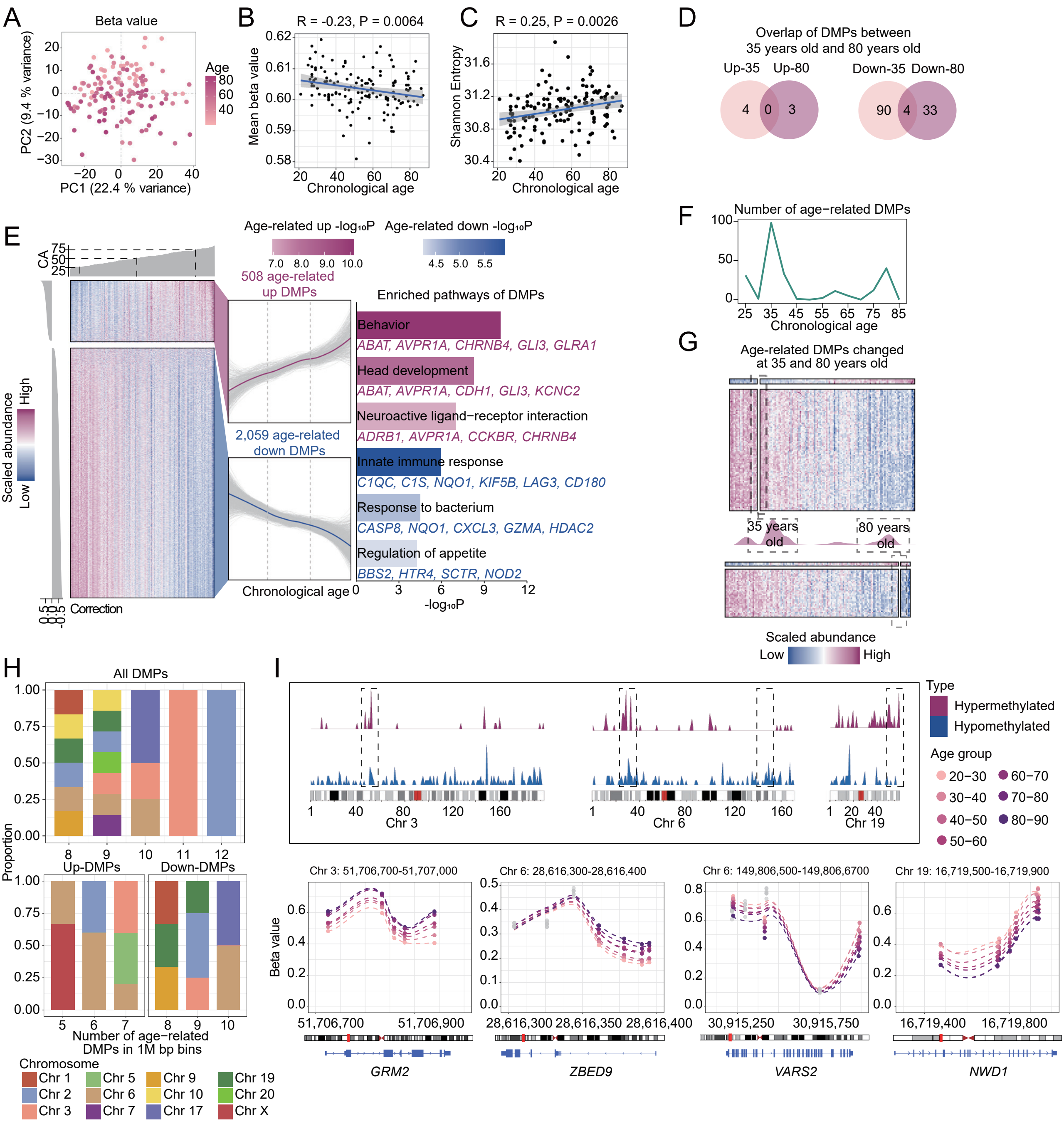

A
Beta value
PC1 (22.4 % variance)
PC2 (9.4 % variance)
Age
B
R = -0.23, P = 0.0064
Mean beta value
Chronological age
C
R = 0.25, P = 0.0026
Shannon Entropy
Chronological age
D
Overlap of DMPs between
35 years old and 80 years old
Up-35
Up-80
Down-35
Down-80
4
0
3
90
4
33
E
Age-related up -log₁₀P
Age-related down -log₁₀P
CA
508 age-related
up DMPs
2,059 age-related
down DMPs
Scaled abundance
High
Low
Correction
Chronological age
Enriched pathways of DMPs
Behavior
ABAT, AVPR1A, CHRNB4, GLI3, GLRA1
Head development
ABAT, AVPR1A, CDH1, GLI3, KCNC2
Neuroactive ligand−receptor interaction
ADRB1, AVPR1A, CCKBR, CHRNB4
Innate immune response
C1QC, C1S, NQO1, KIF5B, LAG3, CD180
Response to bacterium
CASP8, NQO1, CXCL3, GZMA, HDAC2
Regulation of appetite
BBS2, HTR4, SCTR, NOD2
-log₁₀P
F
Number of age−related DMPs
Chronological age
G
Age-related DMPs changed
at 35 and 80 years old
35 years
old
80 years
old
Scaled abundance
Low
High
H
All DMPs
Up-DMPs
Down-DMPs
Proportion
Number of age−related
DMPs in 1M bp bins
Chromosome
Chr 1
Chr 2
Chr 3
Chr 5
Chr 6
Chr 7
Chr 9
Chr 10
Chr 17
Chr 19
Chr 20
Chr X
I
Type
Hypermethylated
Hypomethylated
Age group
20−30
30−40
40−50
50−60
60−70
70−80
80−90
Chr 3
Chr 6
Chr 19
Chr 3: 51,706,700-51,707,000
Chr 6: 28,616,300-28,616,400
Chr 6: 149,806,500-149,806,6700
Chr 19: 16,719,500-16,719,900
Beta value
GRM2
ZBED9
VARS2
NWD1

Fig S4

A

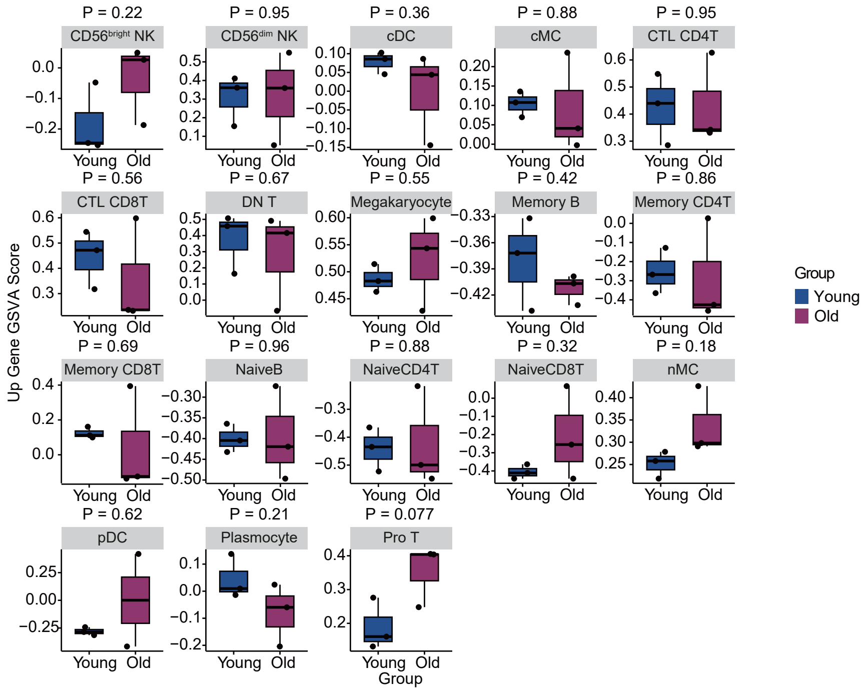

P = 0.22
CD56bright NK
P = 0.95
CD56dim NK
P = 0.36
cDC
P = 0.88
cMC
P = 0.95
CTL CD4T
P = 0.56
CTL CD8T
P = 0.67
DN T
P = 0.55
Megakaryocyte
P = 0.42
Memory B
P = 0.86
Memory CD4T
P = 0.69
Memory CD8T
P = 0.96
NaiveB
P = 0.88
NaiveCD4T
P = 0.32
NaiveCD8T
P = 0.18
nMC
P = 0.62
pDC
P = 0.21
Plasmocyte
P = 0.077
Pro T
Up Gene GSVA Score
Young
Old
Group
Group
Young
Old

B

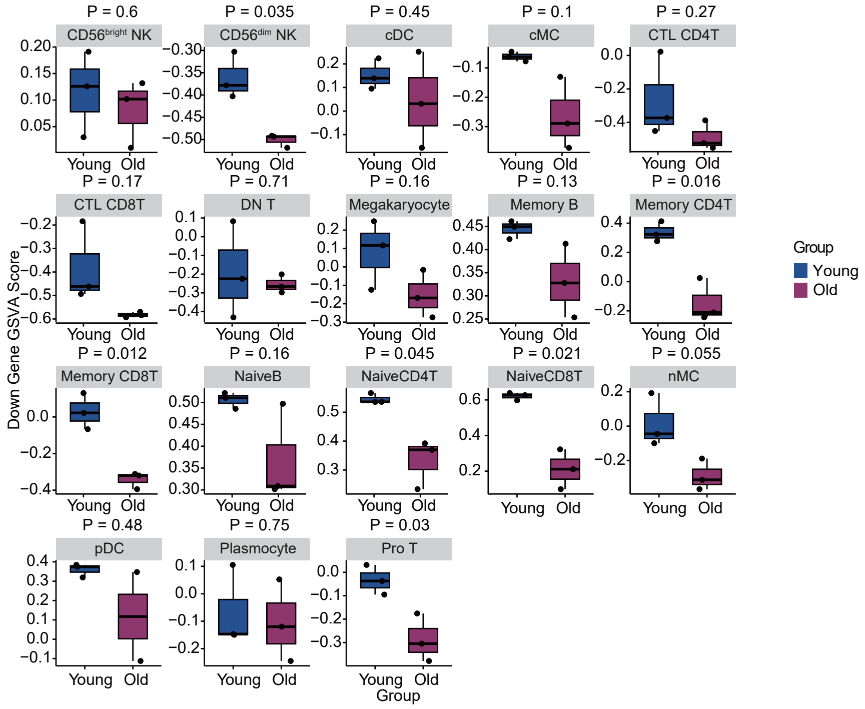

P = 0.6
CD56bright NK
P = 0.035
CD56dim NK
P = 0.45
cDC
P = 0.1
cMC
P = 0.27
CTL CD4T
P = 0.17
CTL CD8T
P = 0.71
DN T
P = 0.16
Megakaryocyte
P = 0.13
Memory B
P = 0.016
Memory CD4T
P = 0.012
Memory CD8T
P = 0.16
NaiveB
P = 0.045
NaiveCD4T
P = 0.021
NaiveCD8T
P = 0.055
nMC
P = 0.48
pDC
P = 0.75
Plasmocyte
P = 0.03
Pro T
Down Gene GSVA Score
Young
Old
Group
Group
Young
Old

Fig S5

A

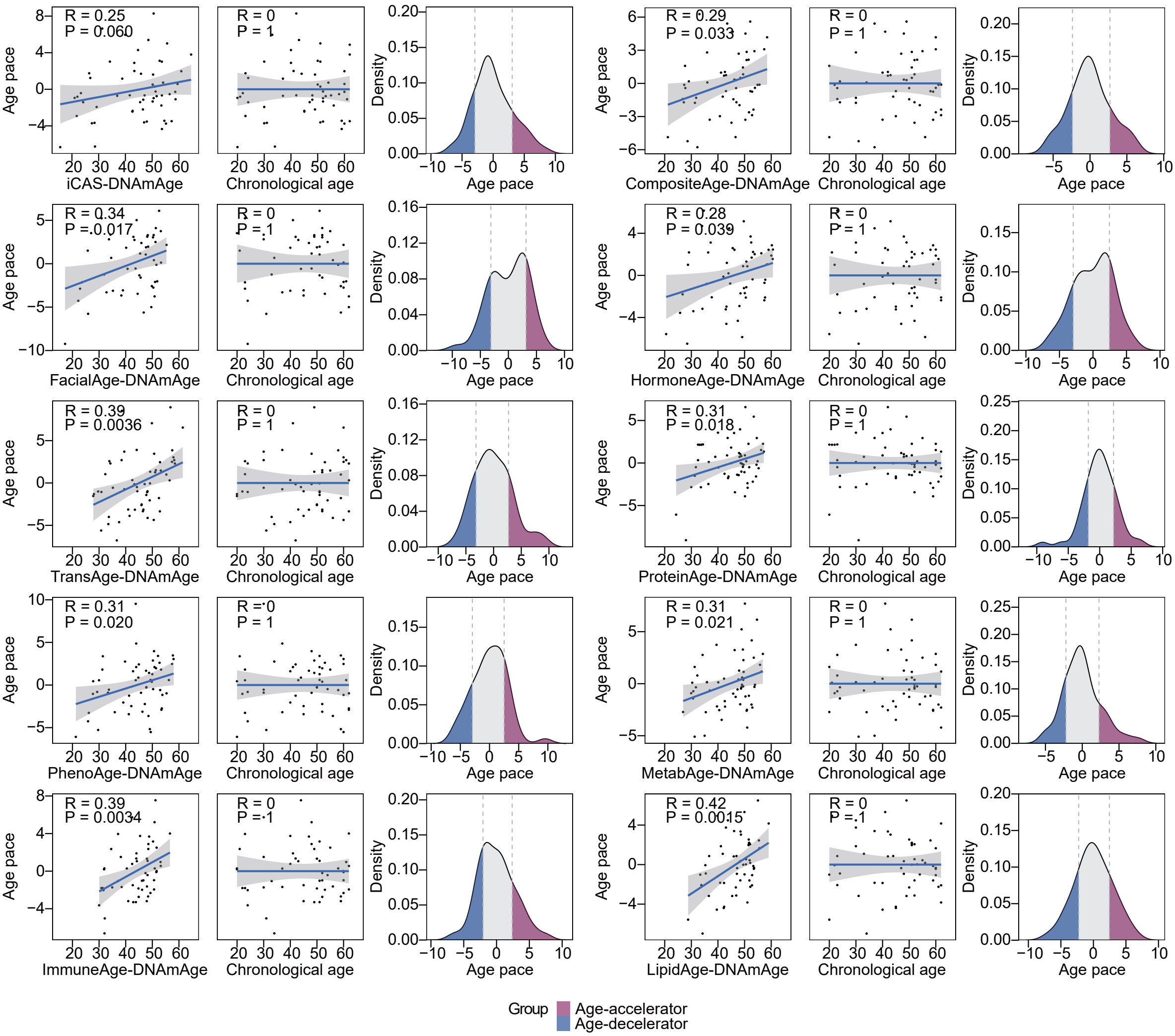
R = 0.25
P = 0.060
iCAS-DNAmAge
R = 0.29
P = 0.033
CompositeAge-DNAmAge
R = 0.34
P = 0.017
FacialAge-DNAmAge
R = 0.28
P = 0.039
HormoneAge-DNAmAge
R = 0.39
P = 0.0036
TransAge-DNAmAge
R = 0.31
P = 0.018
ProteinAge-DNAmAge
R = 0.31
P = 0.020
PhenoAge-DNAmAge
R = 0.31
P = 0.021
MetabAge-DNAmAge
R = 0.39
P = 0.0034
ImmuneAge-DNAmAge
R = 0.42
P = 0.0015
LipidAge-DNAmAge
R = 0
P = 1
Chronological age
Age pace
Density
Group
Age-accelerator
Age-decelerator

Fig S6

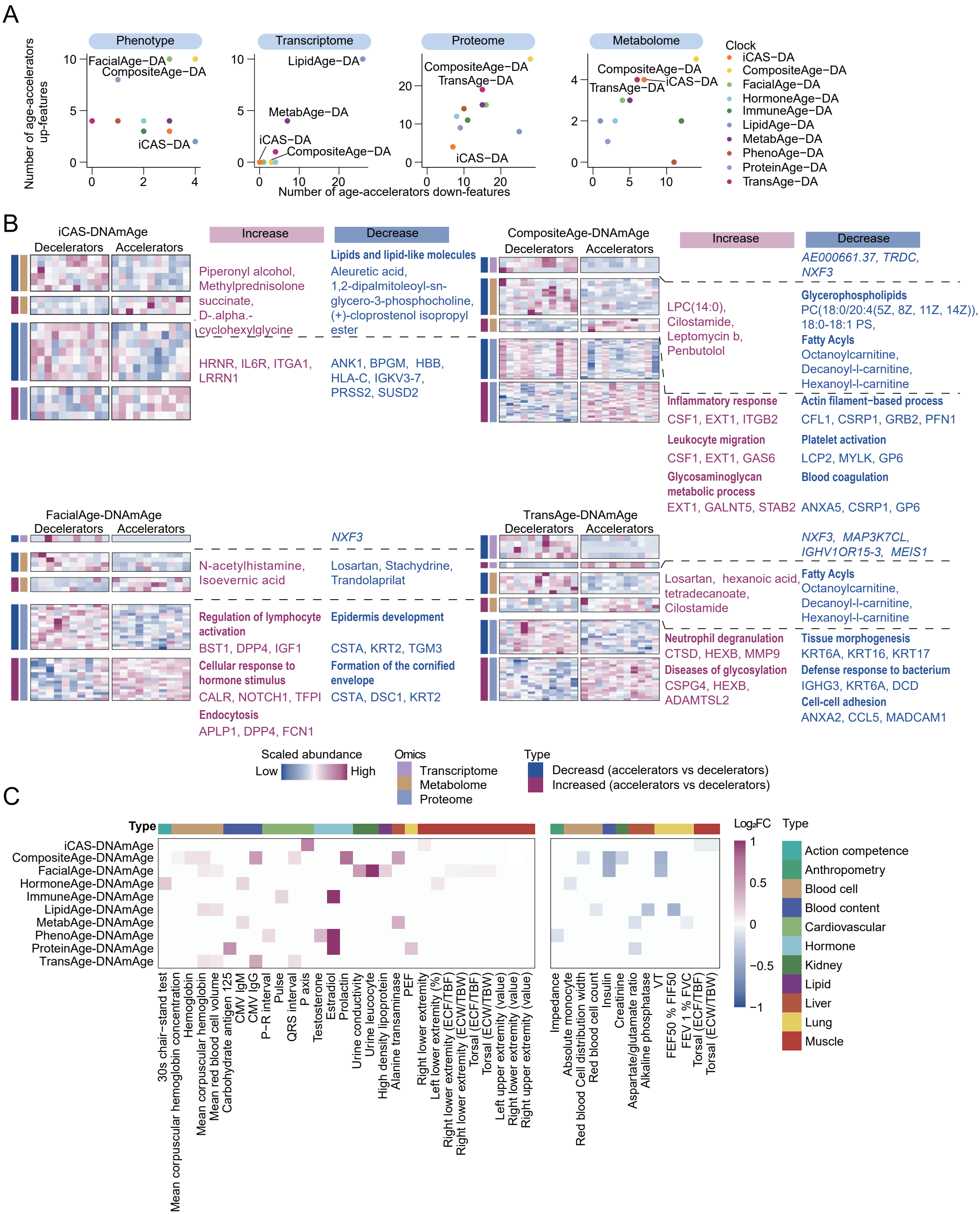

A
Phenotype
Transcriptome
Proteome
Metabolome
Number of age-accelerators up-features
Number of age-accelerators down-features
Clock
iCAS−DA
CompositeAge−DA
FacialAge−DA
HormoneAge−DA
ImmuneAge−DA
LipidAge−DA
MetabAge−DA
PhenoAge−DA
ProteinAge−DA
TransAge−DA
B
iCAS-DNAmAge
Decelerators
Accelerators
Increase
Decrease
Lipids and lipid-like molecules
Piperonyl alcohol, Methylprednisolone succinate, D-.alpha.-cyclohexylglycine
Aleuretic acid, 1,2-dipalmitoleoyl-sn-glycero-3-phosphocholine, (+)-cloprostenol isopropyl ester
HRNR, IL6R, ITGA1, LRRN1
ANK1, BPGM, HBB, HLA-C, IGKV3-7, PRSS2, SUSD2
CompositeAge-DNAmAge
AE000661.37, TRDC, NXF3
LPC(14:0), Cilostamide, Leptomycin b, Penbutolol
Glycerophospholipids
PC(18:0/20:4(5Z, 8Z, 11Z, 14Z)), 18:0-18:1 PS,
Fatty Acyls
Octanoylcarnitine, Decanoyl-l-carnitine, Hexanoyl-l-carnitine
Inflammatory response
CSF1, EXT1, ITGB2
Actin filament−based process
CFL1, CSRP1, GRB2, PFN1
Leukocyte migration
CSF1, EXT1, GAS6
Platelet activation
LCP2, MYLK, GP6
Glycosaminoglycan metabolic process
EXT1, GALNT5, STAB2
Blood coagulation
ANXA5, CSRP1, GP6
FacialAge-DNAmAge
NXF3
N-acetylhistamine, Isoevernic acid
Losartan, Stachydrine, Trandolaprilat
Regulation of lymphocyte activation
BST1, DPP4, IGF1
Epidermis development
CSTA, KRT2, TGM3
Cellular response to hormone stimulus
CALR, NOTCH1, TFPI
Formation of the cornified envelope
CSTA, DSC1, KRT2
Endocytosis
APLP1, DPP4, FCN1
TransAge-DNAmAge
NXF3, MAP3K7CL, IGHV1OR15-3, MEIS1
Losartan, hexanoic acid, tetradecanoate, Cilostamide
Fatty Acyls
Octanoylcarnitine, Decanoyl-l-carnitine, Hexanoyl-l-carnitine
Neutrophil degranulation
CTSD, HEXB, MMP9
Tissue morphogenesis
KRT6A, KRT16, KRT17
Diseases of glycosylation
CSPG4, HEXB, ADAMTSL2
Defense response to bacterium
IGHG3, KRT6A, DCD
Cell-cell adhesion
ANXA2, CCL5, MADCAM1
Scaled abundance
Low
High
Omics
Transcriptome
Metabolome
Proteome
Type
Decreasd (accelerators vs decelerators)
Increased (accelerators vs decelerators)
C
Type
iCAS-DNAmAge
CompositeAge-DNAmAge
FacialAge-DNAmAge
HormoneAge-DNAmAge
ImmuneAge-DNAmAge
LipidAge-DNAmAge
MetabAge-DNAmAge
PhenoAge-DNAmAge
ProteinAge-DNAmAge
TransAge-DNAmAge
30s chair−stand test
Mean corpuscular hemoglobin concentration
Hemoglobin
Mean corpuscular hemoglobin
Mean red blood cell volume
Carbohydrate antigen 125
CMV IgM
CMV IgG
P−R interval
Pulse
QRS interval
P axis
Testosterone
Estradiol
Prolactin
Urine conductivity
Urine leucocyte
High density lipoprotein
Alanine transaminase
PEF
Right lower extremity
Left lower extremity (%)
Right lower extremity (ECF/TBF)
Right lower extremity (ECW/TBW)
Torsal (ECF/TBF)
Torsal (ECW/TBW)
Left upper extremity (value)
Right lower extremity (value)
Right upper extremity (value)
Impedance
Absolute monocyte
Red blood Cell distribution width
Red blood cell count
Insulin
Creatinine
Aspartate/glutamate ratio
Alkaline phosphatase
VT
FEF50 % FIF50
FEV 1 % FVC
Torsal (ECF/TBF)
Torsal (ECW/TBW)
Log2FC
Type
Action competence
Anthropometry
Blood cell
Blood content
Cardiovascular
Hormone
Kidney
Lipid
Liver
Lung
Muscle
